# Mitochondrial dysfunction in immune cells during the perioperative period: mechanisms, emerging therapeutic strategies, and implications for multi-organ protection

**DOI:** 10.3389/fimmu.2026.1734880

**Published:** 2026-03-30

**Authors:** Ziyu Wang, Lu Wang, Maolin Ji, Jie Lou, Yuchuan Yuan, Yuanxu Cui, Gang Xiang, Xing Zhou

**Affiliations:** 1 School of Pharmacy and Bioengineering, Chongqing University of Technology, Chongqing, China; 2 Northwest University, Xi'an, China; 3 Yunnan Key Laboratory of Stem Cell and Regenerative Medicine, Rehabilitation School, Kunming Medical University, Kunming, China

**Keywords:** immune cells, immunomodulation, mitochondrial dysfunction, organ protection, perioperative period

## Abstract

Mitochondrial health is increasingly recognized as a critical determinant of immune competence during the perioperative period. Surgical interventions impose unique metabolic and inflammatory stresses—such as ischemia–reperfusion injury, anesthetic exposure, and systemic inflammatory responses—that impair immune cell bioenergetics and redox balance. Dysfunctional mitochondria in neutrophils, macrophages, and T lymphocytes alter cytokine production, phagocytic activity, and antigen presentation, tipping the balance toward excessive inflammation or postoperative immunosuppression, thereby exacerbating organ injury. This review integrates current knowledge of the mechanisms linking perioperative mitochondrial dysfunction to immune dysregulation, and systematically evaluates emerging therapeutic strategies, including mitochondrial-targeted antioxidants, permeability transition pore inhibitors, metabolic reprogramming agents, mitochondrial transplantation, and gene-based interventions. By bridging experimental evidence with translational and early clinical studies in cardiac, neurological, hepatic, and renal surgeries, we argue that precise modulation of immune cell mitochondrial function represents a promising and underexplored frontier for comprehensive perioperative organ protection.

## Graphical Abstract

**Figure:**
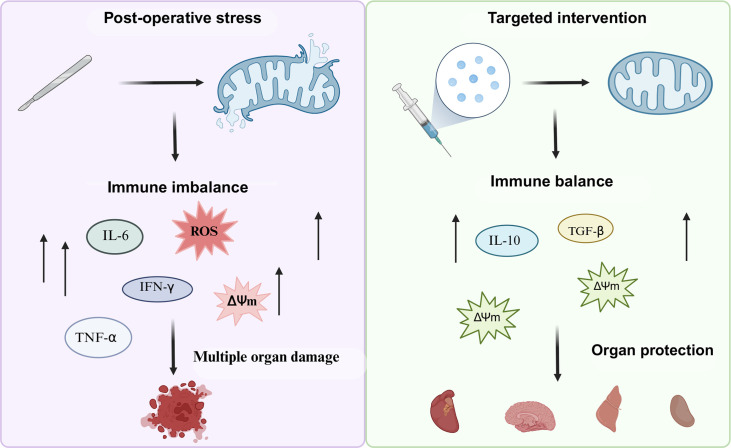

## 1 Introduction

Major surgery and critical illness frequently precipitate a dysregulated immune response that can injure otherwise healthy organs. Despite improvements in surgical techniques and perioperative care, the development of perioperative organ dysfunction – such as acute heart failure, stroke, acute kidney injury (AKI), or liver injury – remains directly associated with worse outcomes and significant mortality (1, 2). Globally, deaths related to postoperative organ failure rank among the top causes of mortality, trailing only cardiovascular disease, cancer, and stroke (2). This clinical reality has galvanized interest in uncovering the underlying mechanisms of surgery-related organ injury and identifying novel preventive therapies.

It is now recognized that excessive inflammation plays a pivotal role in perioperative organ damage (3, 4). This excessive inflammatory response follows a well-characterized temporal trajectory: Systemic Inflammatory Response Syndrome (SIRS), Compensatory Anti-inflammatory Response Syndrome (CARS), and Mixed Antagonist Response Syndrome (MARS). SIRS (postoperative hours to days 2-3) is defined by fever, leukocytosis, and elevated pro-inflammatory cytokines (IL-6, IL-1β, TNF-a). During SIRS, Mitochondrial damage-associated molecular patterns(mtDAMPs activate) TLR9/NLRP3/FPR1 signaling, while reactive oxygen species(ROS)/reactive nitrogen species(RNS) bursts and glycolytic metabolic reprogramming in neutrophils and macrophages fuel rapid ATP generation for antimicrobial defense; unchecked, this drives acute lung injury, AKI, and myocardial dysfunction. CARS (days 3-7) is defined by immune hyporesponsiveness, reduced Human Leukocyte Antigen-DR(HLA-DR) expression, and IL-10/TGF-β predominance. Mitochondrial dysfunction—mpaired oxidative phosphorylation and defective mitophagy—predominates, with metabolic switching to fatty acid oxidation in T cells and M2 macrophage polarization; this immunosuppression increases risks of pneumonia, surgical site infections, and impaired wound healing. MARS represents the dangerous coexistence of persistent SIRS and CARS features, driven by ongoing mtDAMPs release, mitochondrial oxidative stress, and Thymocyte selection-associated high mobility group box protein(TOX)/Nuclear receptor subfamily 4 group A(NR4A)-mediated T cell exhaustion. This state carries the highest mortality risk, as patients experience both refractory organ dysfunction and sepsis from secondary infections (5–7). Surgical tissue injury – whether through direct trauma (incision, retraction), ischemia–reperfusion (IR) during procedures like aortic cross-clamping or organ transplantation, or exposure of blood to foreign surfaces in cardiopulmonary bypass – can trigger a SIRS even in the absence of infection (4). This “sterile” inflammation is driven by endogenous danger signals. During surgery, damaged or ischemic cells release a flood of intracellular contents (so-called damage-associated molecular patterns, DAMPs), which activate the same innate immune receptors that sense pathogens (4). The consequence is a cascade of pro-inflammatory cytokines, complement activation, endothelial dysfunction and coagulopathy that, if unchecked, can culminate in tissue edema, microvascular thrombosis, and multi-organ failure (4). Paradoxically, this initial hyper-inflammatory phase is often followed by a compensatory anti-inflammatory/immunosuppressive phase, rendering patients vulnerable to infections and impaired wound healing – an immune rollercoaster similar to that seen in severe trauma or sepsis.

Mitochondria have emerged as central orchestrators of this perioperative inflammation–immunosuppression continuum. Long appreciated as the “powerhouses” of the cell, mitochondria are now understood to also function as pivotal signaling hubs in immunity (8). Because of their bacterial ancestral origin, mitochondria possess molecules that the immune system perceives as foreign when released extracellularly (9). Chief among these mtDAMPs are mitochondrial DNA (mtDNA) (a circular DNA with unmethylated CpG motifs akin to bacterial DNA), N-formyl peptides (derivatives of mitochondrial proteins initiating with N-formyl-methionine, mimicking bacterial peptides), the phospholipid cardiolipin from the inner mitochondrial membrane, and byproducts like ATP and ROS. Normally sequestered within mitochondria, these molecules are released during cell necrosis or mitochondrial stress and can potently activate immune cells (10). For example, cell-free mtDNA is sensed by TLR9 in endosomes (recognizing CpG DNA) and by Cyclic GMP-AMP synthase (cGAS) in the cytosol (recognizing DNA in general), leading to production of inflammatory cytokines and interferons (11–14). Likewise, extruded ATP acts on purinergic receptors (like P2X7) to assemble the NLRP3 inflammasome complex, while oxidized mtDNA directly binds and activates NLRP3 inside macrophages (15). Neutrophils and monocytes express formyl peptide receptor-1 (FPR1) that detects N-formyl peptides, triggering chemotaxis and activation similar to sensing bacteria (16, 17). In essence, mtDAMPs erroneously “alert” the immune system to a severe infection, when in fact the trigger is trauma or hypoxia from surgery – thus amplifying sterile inflammation.

Concurrently, mitochondrial dysfunction within immune cells themselves critically shapes the quality of the immune response. Immune cell activation and phenotype are tightly linked to cellular metabolism and mitochondria (a field known as immunometabolism). Surgical stress can impose drastic shifts in immune cell metabolism – for instance, a switch from aerobic respiration to glycolysis – that affect immune cell function (6). Studies have shown that in sepsis and major trauma, persistent mitochondrial dysfunction in circulating immune cells correlates with immune paralysis and worse outcomes (18). Key immune functions like phagocytosis, antigen presentation, and lymphocyte proliferation all depend on adequate ATP supply and mitochondrial signaling; when mitochondria are impaired (*e.g.*, by oxidative damage or membrane depolarization), immune cells may become either hyper-inflammatory (dumping toxic radicals, extracellular traps, *etc.*) or anergic (unable to mount responses) (19). For example, neutrophils with damaged mitochondria show reduced bactericidal activity and a propensity for excessive neutrophil extracellular trap (NET) formation, contributing to tissue injury (20). Macrophages with blocked mitochondrial respiration remain stuck in an M1 pro-inflammatory state and fail to resolve inflammation or transition to healing phenotypes (21). T-cells require a metabolic shift to glycolysis for clonal expansion but then rely on robust mitochondrial oxidative metabolism for memory and sustained function; major surgery can blunt this metabolic flexibility, leading to T-cell exhaustion or apoptosis (22, 23).

Considering these dual roles of mitochondria – as instigators of innate immune activation *via* DAMPs release, and as regulators of immune cell fate *via* metabolism – it is logical that therapeutically modulating mitochondrial function could have profound benefits for perioperative organ protection. This concept has spurred a new wave of research at the intersection of mitochondrial biology and perioperative medicine. Investigators are examining interventions ranging from pharmacological agents (*e.g.*, mitochondria-targeted antioxidants, metabolic modulators) to biologic therapies (*e.g.*, cytokine inhibitors, peptides that stabilize mitochondria) to cellular/gene therapies (*e.g.*, transfusing healthy mitochondria or engineering patient immune cells for improved mitochondrial performance). Early findings are encouraging – for instance, selectively inhibiting the NLRP3 inflammasome or scavenging circulating mtDNA in animal models markedly attenuates IR-induced organ damage (24). Novel therapies like mitochondrial transplantation have shown striking improvements in heart and lung function after ischemic injury in preclinical studies (25–27). Moreover, retrospective analyses in surgical patients hint that some existing practices may exert mitochondria-mediated protective effects: *e.g.*, use of volatile anesthetic agents and opioids can precondition the heart by modulating mitochondrial potassium channels and reducing ROS generation (28); or maintaining normothermia avoids hypothermia-induced mitochondrial oxidative stress (29).

**Figure 1:**
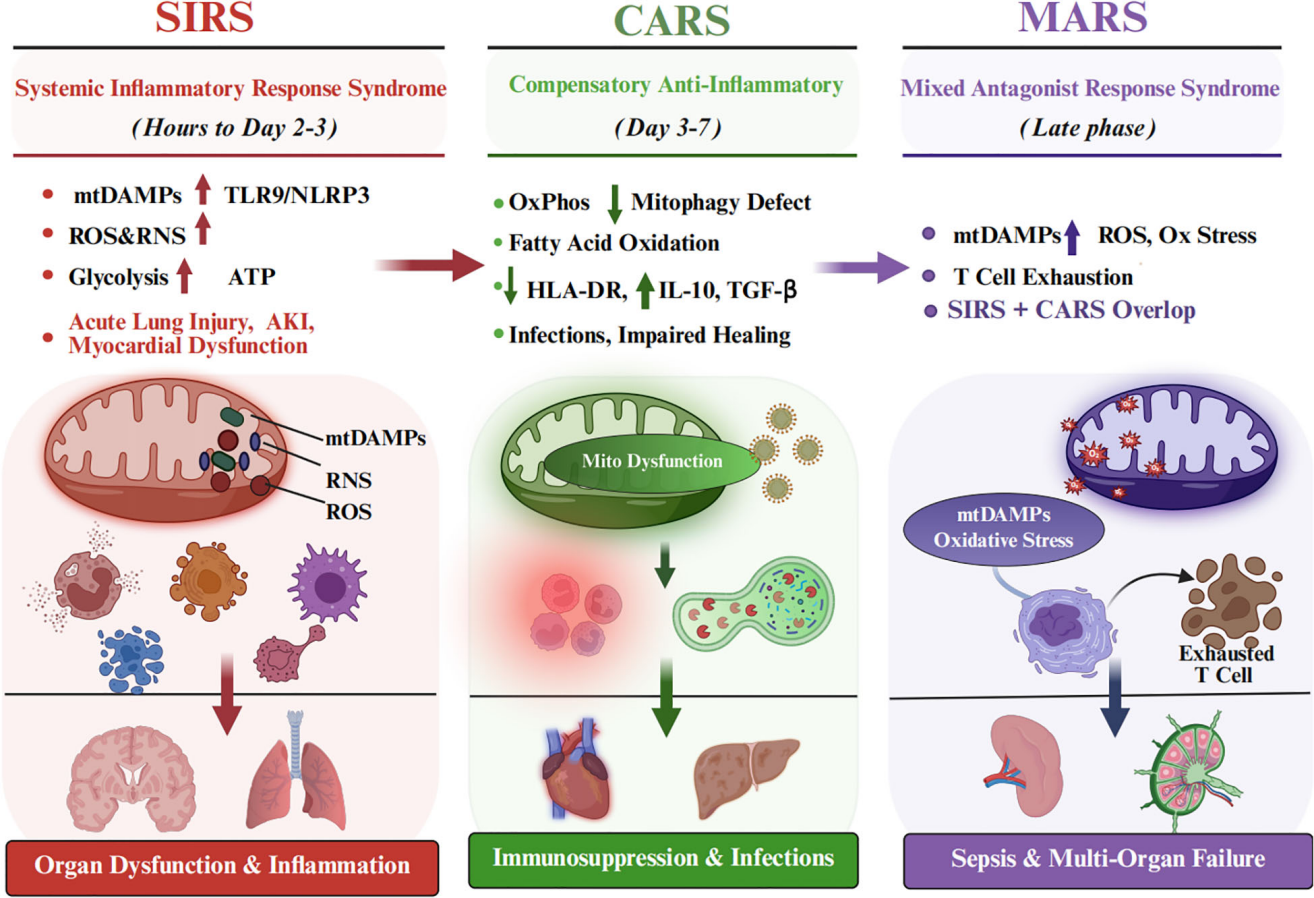
The SIRS–CARS–MARS continuum of perioperative immune dysregulation driven by mitochondrial stress.

While excellent recent reviews have summarized mtDAMP biology in sterile inflammation and systemic diseases (30–32), the present work offers distinctive contributions tailored to the perioperative context by establishing the perioperative continuum—from preoperative optimization through intraoperative IR injury to postoperative recovery—as a unified temporal framework for mitochondrial intervention, moving beyond isolated disease models; by forging mechanistic-translational bridges that explicitly connect specific surgical insults (cardioplegic arrest, aortic cross-clamping, hepatic ischemia) to defined nodes of the IR-mtDAMP axis; by systematically evaluating pharmacologic, biologic, and engineering strategies specifically for surgical applicability; and by introducing clinically actionable decision nodes—themitochondrial permeability transition pore (mPTP) “safe return point” and TOX/NR4A exhaustion checkpoint—that translate mitochondrial biology into anesthesia and critical care decision-making (30–32)., our review focuses on the perioperative setting. We organize mitochondrial stress and mtDAMP signaling along the surgical time course, from preoperative risk/metabolic status, through intraoperative IR, to postoperative immune recovery. We also relate common surgical insults (e.g., cardioplegic arrest/cardiopulmonary bypass, aortic cross-clamping, hepatic inflow occlusion) to specific nodes of the IR–mtDAMP axis and downstream inflammatory pathways. Finally, we discuss organ-protection strategies with explicit attention to perioperative feasibility and timing, including pharmacologic approaches, biologics and emerging bioengineering interventions. Where evidence is limited, we outline testable perioperative readouts and windows—such as reperfusion-linked mPTP susceptibility and TOX/NR4A-associated T-cell dysfunction programs—to support patient stratification and trial design. The SIRS-CARS-MARS continuum of perioperative immune dysregulation driven by mitochondrial stress is illustrated in **Figure 1**.

In this comprehensive review, we synthesize current knowledge of how perioperative immune cell mitochondrial dysfunction contributes to organ injury and detail the emerging mitochondria-centric interventions aiming to improve outcomes. We begin with the mechanistic basis: how surgical stress injures mitochondria and how in turn dysfunctional mitochondria dysregulate immune responses. We then discuss targeted therapeutic strategies – spanning drugs, biologics, and gene/cell therapies – that seek to protect or restore immune cell mitochondrial function in the perioperative period. Next, we review clinical evidence from cardiac, neurologic, and abdominal surgery contexts, highlighting both correlative studies (*e.g.*, biomarkers like mtDNA and outcomes) and interventional trials (*e.g.*, trials of anti-inflammatory or metabolic therapies). Finally, we address challenges and future directions, including patient stratification by immunometabolic status, safety considerations of mitochondria-targeted treatments, and opportunities to integrate mitochondrial monitoring into perioperative care. By bridging these insights, we aim to elucidate why modulating immune cell metabolism and mitochondria is a promising new frontier and how it could transform organ protection in surgery – moving beyond generalized anti-inflammatory approaches towards precise immunometabolic therapy that DAMPens harmful inflammation without crippling host defenses.

## 2 Immune cell mitochondrial dysfunction mechanisms in the perioperative period

**Figure 2:**
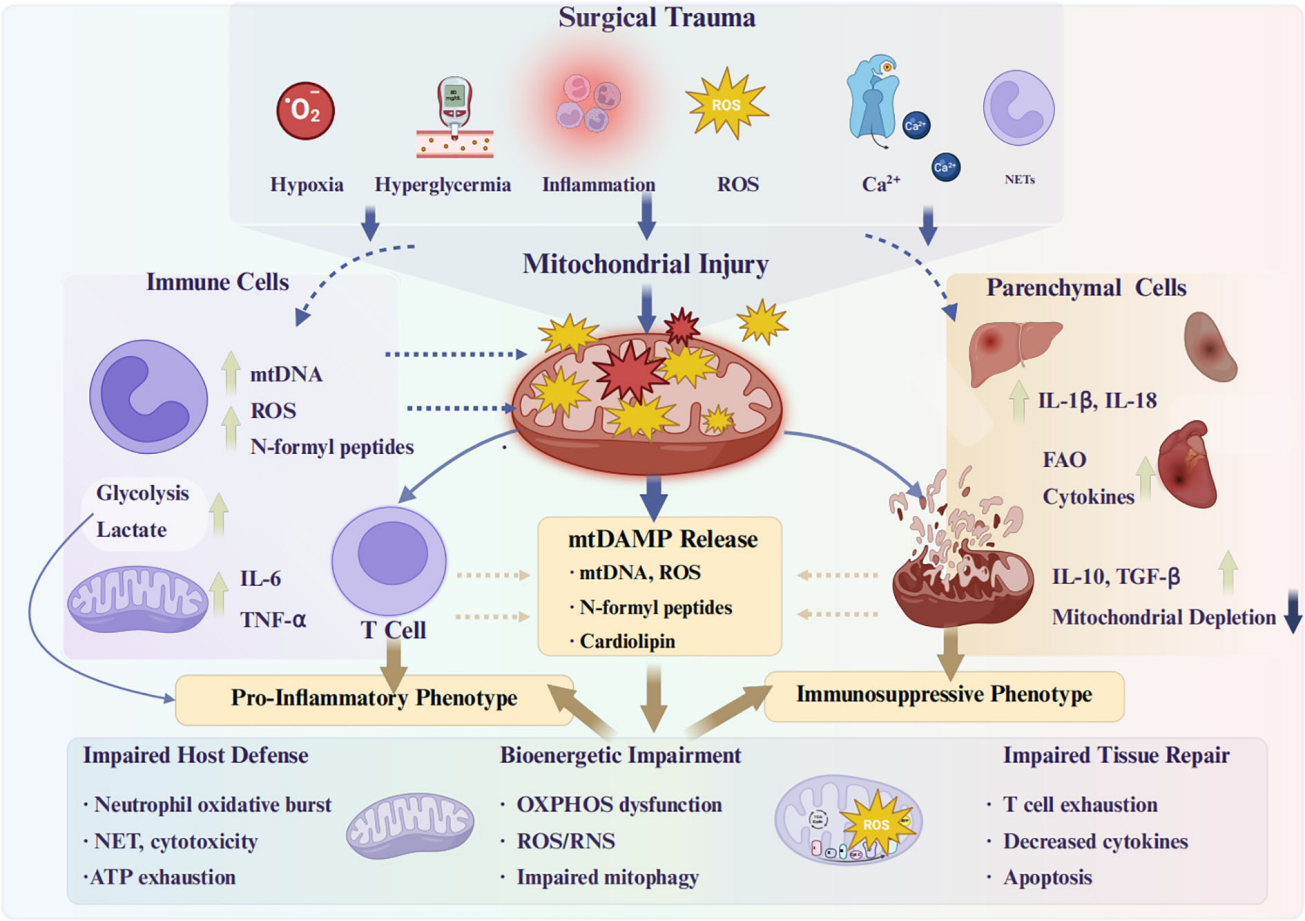
Mechanistic overview of mitochondrial dysfunction in immune cells during the perioperative period.

The perioperative period represents a convergence of multiple physiological stressors—including tissue ischemia–reperfusion, mechanical injury, anesthesia exposure, and neuroendocrine surges—that collectively challenge immune cell homeostasis at the mitochondrial level. Mitochondria, as both energy generators and innate immune signaling hubs, are uniquely positioned to determine the trajectory of postoperative immune responses. Surgical trauma not only precipitates structural damage to mitochondria in immune and parenchymal cells, but also perturbs their metabolic programming, redox balance, and quality-control mechanisms. These perturbations lead to the release of mtDAMPs, rewire immune cell metabolism toward pro-inflammatory or immunosuppressive phenotypes, and impair the bioenergetic capacity needed for effective host defense and tissue repair (**Figure 2**). Understanding the mechanistic interplay between mitochondrial injury, immune activation, and metabolic reprogramming provides a unifying framework for explaining perioperative complications and identifying targeted interventions to preserve organ function.

### 2.1 mtDAMPs release and innate immune activation

#### 2.1.1 Tissue injury and mitochondrial danger signals

During surgery and acute trauma, abrupt tissue damage and hypoxia lead to cellular necrosis, releasing not only cytosolic contents but also mitochondrial constituents into the circulation. Mitochondria, due to their bacterial origin, carry molecules that act as DAMPs “red flags” to the immune system (9, 10). This process is analogous to an infection, except the source of the “pathogen” signals is the patient’s own damaged cells. Key mitochondrials implicated in postoperative inflammation include mtDNA, formylated peptides, cardiolipin, mitochondrial ATP, and mitochondrial ROS:

##### 2.1.1.1 mtDNA

Perhaps the most potent DAMPs, mtDNA is normally confined within mitochondria. Surgery involving cardiopulmonary bypass (CPB) or major trauma can cause a massive surge of cell-free mtDNA in plasma – often rising by an order of magnitude during or shortly after surgery. For example, in a study of patients undergoing cardiac surgery with CPB, circulating mtDNA increased ~16-fold by the end of bypass (33). Similarly, a rodent model of surgical trauma showed a 19-fold rise in plasma mtDNA within minutes of incision (34). This mtDNA can be released from shattering cells or actively extruded by stressed cells (*e.g.*, *via* extracellular vesicles or neutrophil NETs). Once in the extracellular space, mtDNA is recognized by TLR9 in endosomes of dendritic cells, macrophages, and B-cells, triggering MyD88-dependent NF-κB activation and a burst of pro-inflammatory cytokines like IL-6, TNF-α.

In the cytosol, mtDNA activates cyclic GMP–AMP synthase–stimulator of interferon genes pathway(the cGAS-STING pathway) to induce type I interferons and enhance NF-κB signaling (35, 36). Oxidized mtDNA has been shown to strongly activate NLRP3 inflammasomes inside macrophages (37, 38). Thus, mtDNA is a versatile that engages multiple inflammatory pathways (TLR9, cGAS-STING, NLRP3). Clinically, higher postoperative mtDNA levels have been associated with increased IL-6, IL-8, and C-reactive protein (CRP) levels, more postoperative complications, and even cognitive dysfunction (12–14). One study identified cell-free mtDNA as a candidate biomarker for predicting patients at risk of SIRS and organ dysfunction after cardiac surgery (33).

##### 2.1.1.2 N-formyl peptides

These are short peptides originating from mitochondrial proteins that still initiate with N-formyl-methionine (a signature of bacteria and mitochondria). Neutrophils and other phagocytes have formyl peptide receptors (FPR1, FPR2) that bind N-formyl peptides. In trauma patients, mitochondrial N-formyl peptides released into the blood have been shown to activate neutrophils and cause chemotaxis and capillary leak syndrome similar to bacterial sepsis (39). They essentially trick neutrophils into “chasing a phantom infection.” Unchecked, this can contribute to distant organ injury (*e.g.*, neutrophil sequestration in lungs causing acute respiratory distress syndrome, ARDS). Antagonists of FPR1 are being explored to mitigate excessive neutrophil activation in sterile inflammation.

##### 2.1.1.3 Cardiolipin

An inner mitochondrial membrane phospholipid, cardiolipin externalization to the outer membrane is a sign of mitochondrial damage. Exposed cardiolipin can directly bind to the NLRP3 inflammasome complex and promote its activation (40, 41). It effectively acts as a platform for NLRP3 assembly on mitochondria. Studies show that disruption of cardiolipin synthesis or preventing cardiolipin oxidation suppresses NLRP3 activation (41). During surgery, oxidative stress can oxidize cardiolipin; oxidized cardiolipin not only triggers NLRP3 but also signals for mitophagy (mitochondrial autophagy) as a quality control. If mitophagy fails, the accumulation of cardiolipin on damaged mitochondria can keep inflammasomes turned on (15). Cardiolipin may also contribute to the formation of neutrophil extracellular traps. Thus, cardiolipin is another mitochondrial “alarm” molecule connecting mitochondrial damage to inflammation.

##### 2.1.1.4 Mitochondrial ROS and ATP

IR and surgical stress lead to bursts of mitochondrial ROS. Excess ROS not only damages tissues but also serves as a second messenger for immune activation – for instance, ROS can activate NF-κB and stabilize HIF-1α in immune cells, skewing macrophages to a pro-inflammatory phenotype (42). ROS is also required for optimal NLRP3 inflammasome activation (37, 38). Meanwhile, extracellular ATP, when released from traumatized cells or leaking mitochondria, binds purinergic P2X7 receptors on macrophages and dendritic cells, leading to K^+^ efflux and NLRP3 inflammasome assembly, causing maturation of IL-1β and IL-18 (24). High ATP levels in the damaged tissue microenvironment thus propagate inflammatory signaling. Notably, surgical patients often have elevateitochondrial d ATP and adenosine levels locally, which modulate immune cell recruitment and cytokine profiles (40).

#### 2.1.2 Activation of innate immune pathways

**Figure 3:**
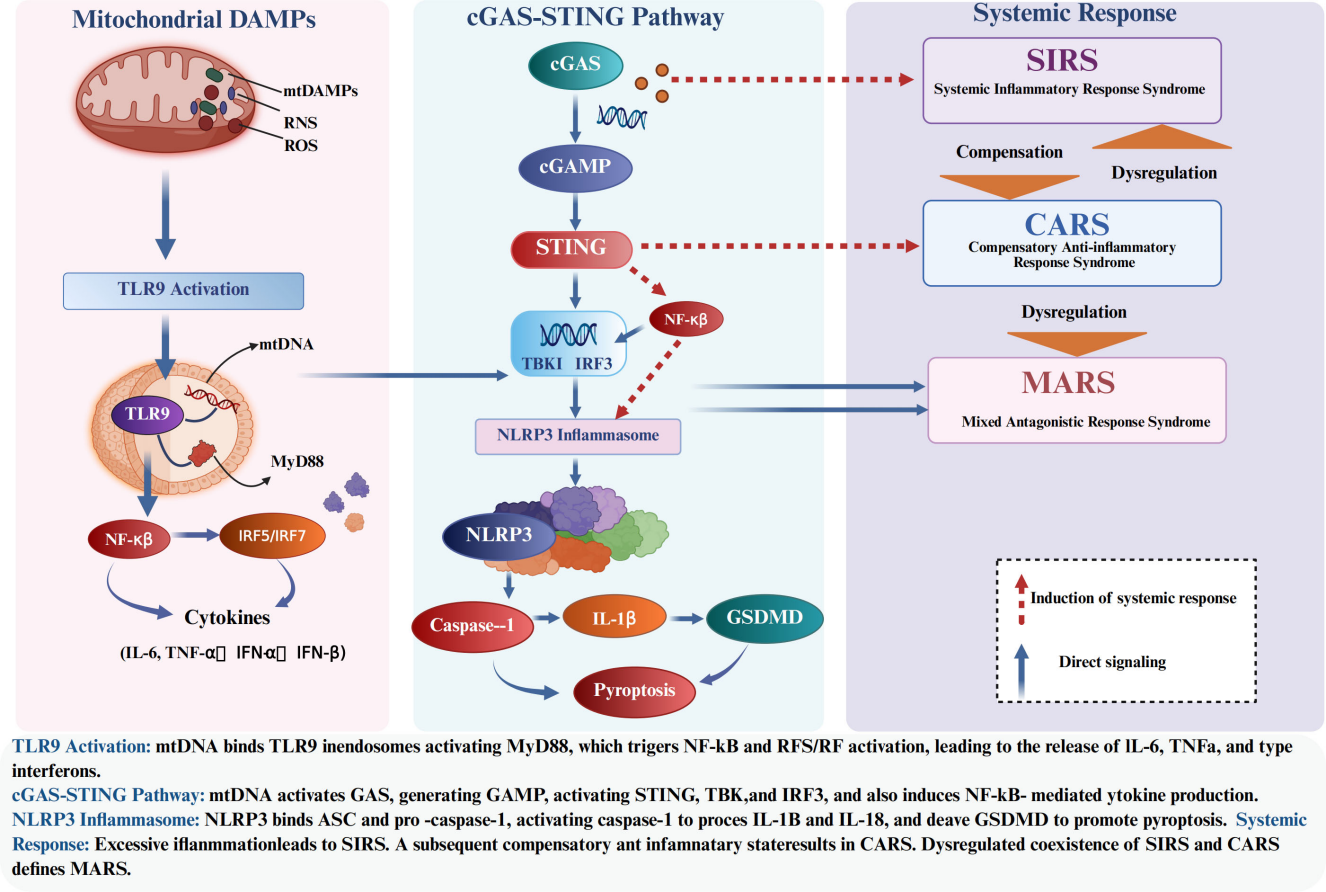
Mitochondrial DAMP-Induced Inflammatory Pathways and Systemic Response.

The mDAMPs discussed converge on specific innate immune pathways. **Figure 3** summarizes the major inflammatory pathways activated by mitochondrial DAMPs, including TLR9, NLRP3, and cGAS-STING signaling, and their systemic consequences.

##### 2.1.2.1 TLR9/MyD88 pathway

**Figure 4:**
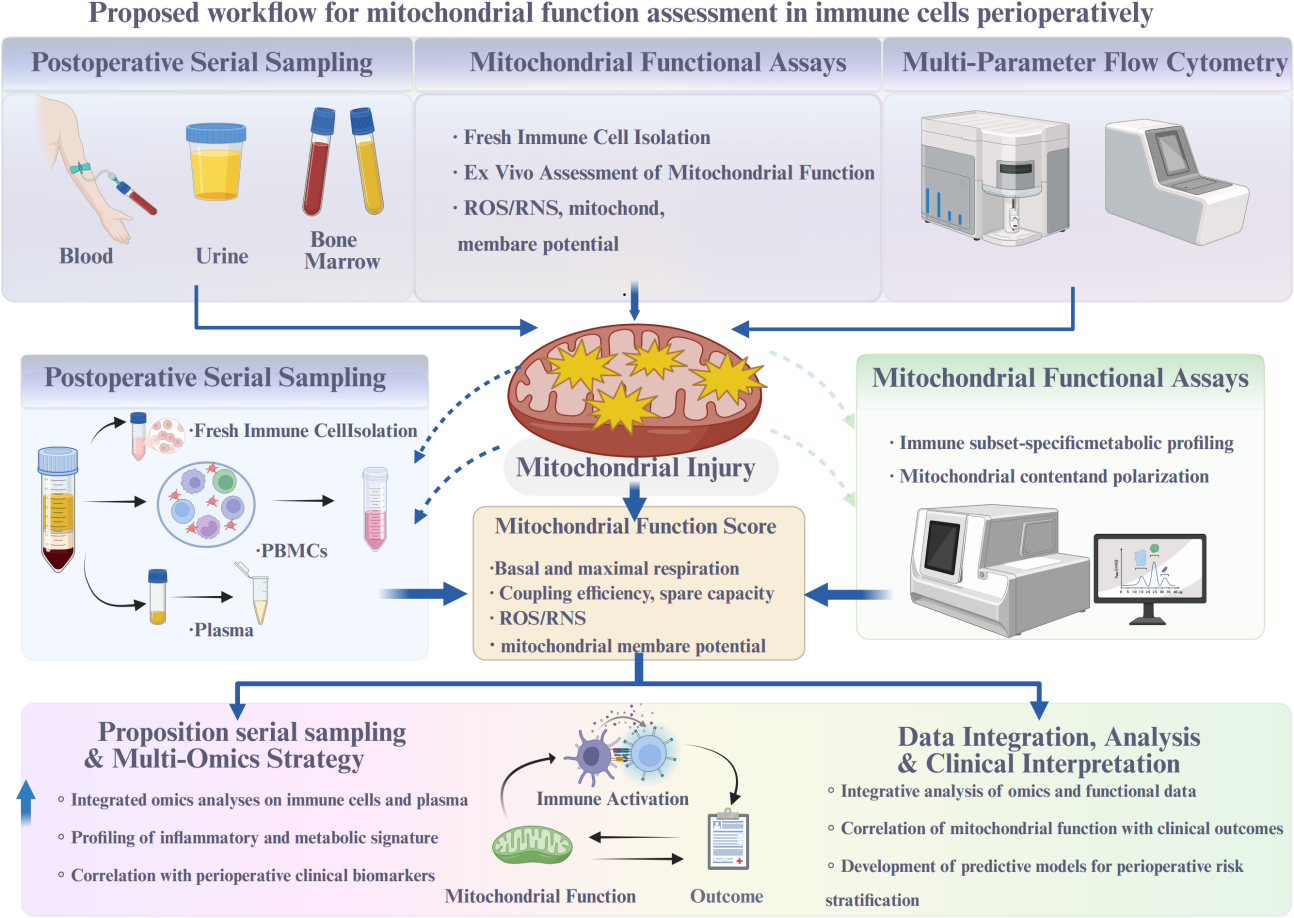
Proposed workflow for mitochondrial function assessment in immune cells perioperatively.

TLR9 senses mtDNA and drives inflammatory responses in surgical settings (34), TLR9 knockout mice are protected from some forms of sterile organ injury. In humans, studies of cardiac surgery patients show a correlation between TLR9 activation (measured by downstream cytokines) and postoperative complications (33). Its downstream MyD88 signaling leads to NF-κB nuclear translocation and induction of IL-1, IL-6, TNF, and chemokines that recruit more immune cells (34). This can create a vicious cycle: inflammation -> tissue damage -> more mitochondrial release -> more inflammation (**Figure 4**).

##### 2.1.2.2 NLRP3 inflammasome

NLRP3 is a cytosolic immune sensor that responds to a variety of danger signals (K^+^ efflux, ROS, DNA, lysosomal rupture). Mitochondrial damage is a well-known activator of NLRP3 (37, 38). Mechanistically, mitochondrial ROS and oxidized mtDNA released into the cytosol bind to NLRP3, and cardiolipin on damaged mitochondria helps cluster NLRP3 with its adaptor ASC and pro-caspase-1 (43). This leads to caspase-1 activation, which cleaves pro-IL-1β and pro-IL-18 into their active, highly inflammatory forms. In Kupffer cells (liver macrophages) during liver IR, it’s shown that NLRP3-driven IL-1β release contributes to hepatocyte injury and neutrophil recruitment (24). Inhibition of NLRP3 (*e.g.*, with the small molecule MCC950) or knocking out NLRP3 confers protection in models of myocardial IR, cerebral IR, and others (24). Of note, a feed-forward loop may occur whereby IL-1β itself triggers further mtDNA release, sustaining inflammation (44, 45). Thus, NLRP3 inflammasome acts as a master amplifier of sterile inflammation once mitochondria are damaged.

##### 2.1.2.3 cGAS-STING pathway

The cGAS-STING DNA-sensing pathway is activated in sterile injury. During ischemia-reperfusion, both nuclear and mitochondrial DNA can leak out of cells or into the cytosol. Animal studies show robust activation of STING in heart, brain, and kidney after IR injury, and genetic or pharmacologic inhibition of STING reduces tissue damage and inflammation (46, 47). In liver transplantation models, blocking mtDNA release (using DNase or Bax/Bak inhibitors to prevent mitochondrial membrane permeabilization) ameliorated injury by avoiding cGAS-STING activation (48). Activation of STING leads to TBK1 and IRF3 phosphorylation, inducing interferons and many interferon-stimulated genes, as well as NF-κB activation (49, 50).Notably, circulating mtDNA in surgical patients has been correlated with STING pathway activation markers and with endothelial dysfunction (51, 52). This implicates the cGAS-STING axis in postoperative organ injuries such as lung injury and AKI.

##### 2.1.2.4 Inflammasome-independent IL-33 and HMGB1 release

Mitochondrial damage can also prompt stressed cells (*e.g.*, endothelial or epithelial cells) to release alarmins like IL-33 or HMGB1. HMGB1 (mainly nuclear, but also mitochondrial-binding) is a late mediator of inflammation that is elevated post-surgery and contributes to organ dysfunction. Mitochondrial ROS can cause HMGB1 translocation and secretion. IL-33, an “alarmin” cytokine stored in nuclei, is released upon cell damage and has complex effects (can be pro-inflammatory or tissue-protective). These factors extend the network of DAMPs signaling beyond the classical receptors and can further modulate immune cell responses in the postoperative period (for example, IL-33 can drive type 2 immunity and might aid in resolution of injury, whereas HMGB1 sustains inflammation and also promotes immunosuppression in late phases) (53). Therapeutically, neutralizing HMGB1 or blocking its receptor (RAGE/TLR4) has shown protection in preclinical sepsis and could be relevant in surgical SIRS as well.

In summary, the perioperative innate immune response is heavily influenced by mtDAMPs release. Tissue injury unleashes mtDNA, formyl peptides, and other mitochondrial signals into the circulation, which engage pattern recognition receptors like TLR9, NLRP3, and STING in immune cells. The result is an acute inflammatory storm – high levels of IL-1β, IL-6, TNF-α, IFN-α/β, *etc.* – that can cause collateral damage to organs. This helps explain why patients with extensive surgical trauma or prolonged ischemia often develop a systemic inflammatory response that parallels septic shock, even in the absence of infection (54). The severity of this initial inflammatory hit often correlates with downstream organ injury (for instance, peak IL-6 after cardiac surgery is a strong predictor of postoperative atrial fibrillation and AKI). It also sets the stage for subsequent immune dysfunction.

Importantly, these pathways offer targets for intervention: blocking upstream mtDAMPs release or downstream receptors could blunt the harmful inflammation. Proof of principle has been shown in experimental models: *e.g.*, pharmacologic inhibition of TLR9 (with IRS-954 oligodeoxynucleotide) reduced IL-6 by >90% and mitigated kidney IR injury in mice; NLRP3 inhibitor MCC950 lessened myocardial infarct size in mice and reduced inflammatory cell infiltration; STING inhibitors (like H-151) protected mice from cerebral IR injury with less microglial activation and neuronal death. These findings will be revisited in the Therapeutic Strategies section, but mechanistically they underscore that DAMPsening the mtDAMPs-innate receptor axis is a viable approach to reduce surgical organ damage.

#### 2.1.3 Metabolic reprogramming and dysfunction in immune cells

Beyond the triggering of innate immune receptors, the perioperative environment profoundly affects immune cell metabolism, which in turn alters immune cell function. Immune responses are energetically demanding – from neutrophils generating an oxidative burst to T-cells proliferating – and thus rely on dynamic mitochondrial support (**Figure 2**). Surgical stressors like hypoxia, hyperglycemia, catecholamine surges, and inflammatory mediators can all perturb immune cell metabolism.

Several specific mechanisms of immune cell mitochondrial dysfunction in the perioperative period have been identified:

##### 2.1.3.1 Warburg-like effect in innate immune cells

The Warburg effect, first described by Otto Warburg in the 1920s, refers to the phenomenon wherein cancer cells preferentially metabolize glucose via aerobic glycolysis rather than oxidative phosphorylation, even in the presence of adequate oxygen (55). In malignancy, this constitutes a constitutive, irreversible metabolic adaptation driven by oncogenic signaling (e.g., HIF-1α stabilization, c-Myc amplification) that supports uncontrolled proliferation independent of oxygen availability. However, aerobic glycolysis in activated immune cells represents a fundamentally distinct biological process. The glycolytic switch observed in macrophages and T cells is a tightly regulated, reversible transition precisely calibrated to meet dynamic effector demands. This immunometabolic reprogramming is governed by signaling pathways (mTOR, AMPK, HIF-1α) that respond to acute environmental cues rather than genetic transformation, and rapidly reverts to oxidative phosphorylation upon stimulus withdrawal (56, 57).

This distinction has profound functional consequences for immune cell biology. Effector T cells transiently adopt high glycolytic flux to support rapid proliferation, cytokine synthesis (notably IFN-γ production requires glycolytic intermediates), and migration to inflammatory sites. Conversely, memory T cells rely predominantly on fatty acid oxidation and oxidative phosphorylation to ensure long-term survival and rapid recall capacity. Similarly, macrophage polarization exemplifies regulated immunometabolism: pro-inflammatory M1 macrophages exhibit high glycolytic activity to support iNOS-mediated NO production, whereas anti-inflammatory M2 macrophages depend on oxidative phosphorylation for tissue repair functions (58, 59). Perioperative IR injury and persistent mtDAMPs exposure may disrupt this metabolic flexibility, locking cells in exhausted states with impaired function. Critically, perioperative metabolic reprogramming is driven by acute DAMPs/mtDAMPs exposure, ischemia-reperfusion-induced hypoxia/reoxygenation, and the inflammatory cytokine milieu—not by oncogenic transformation. Explicit acknowledgment of these distinctions prevents inappropriate extrapolation from tumor biology to immunology and accurately reflects the reversible, signal-dependent nature of immunometabolism in surgical contexts (60).

##### 2.1.3.2 Mitochondrial respiratory dysfunction and ROS overload

In trauma and major surgery, studies of circulating leukocytes (*e.g.*, using high-resolution respirometry) have found reduced mitochondrial oxygen consumption rates and membrane depolarization, indicating intrinsic mitochondrial dysfunction (61). Causes include damage to electron transport chain complexes by ROS and nitric oxide-derived species, calcium overload in mitochondria during stress, and downregulation of mitochondrial biogenesis pathways in immune cells. Neutrophils from postoperative patients often exhibit decreased spare respiratory capacity and increased production of ROS at baseline (62). This means neutrophils may paradoxically cause tissue oxidative damage while being less effective at killing microbes (since controlled ROS burst is needed inside phagosomes, but uncontrolled ROS outside causes tissue injury). Oxidative stress can also trigger premature neutrophil apoptosis or, conversely, NETosis (expulsion of nuclear chromatin webs), both of which can worsen outcomes – NETs, for instance, have been implicated in postoperative organ failure like TRALI (transfusion-related lung injury) and microthrombosis (63). In macrophages, damaged mitochondria continuously leak ROS and even release mtDNA into the cytosol, thus self-amplifying inflammasome activation (37, 38). If mitophagy (mitochondrial autophagy) is impaired – as can occur due to inflammatory signaling or aging – these dysfunctional mitochondria accumulate. One study noted that after surgical stress, monocytes had increased markers of mitochondrial damage but reduced expression of PINK1 and Parkin (key mitophagy proteins), suggesting defective clearance of bad mitochondria (64). Enhancing mitophagy in such cells (experimentally *via* PINK1 overexpression) could reduce ROS and inflammasome activity, highlighting the tight link between mitochondrial quality control and immune cell inflammatory status (65).

##### 2.1.3.3 Immune cell exhaustion and energy failure: T-lymphocytes

(especially CD4^+^ and CD8^+^ T cells) are highly sensitive to metabolic perturbations. After major surgery, patients often exhibit transient T-cell immunosuppression – for example, reduced IL-2 production and proliferative anergy in response to mitogens, contributing to increased infection risk in the week following surgery (64, 66). A contributor to this is T-cell “exhaustion,” similar to states seen in chronic infections and cancer. One mechanism is *via* inhibitory receptors like PD-1 being upregulated in the stress environment; PD-1 signaling in T-cells was shown to suppress PGC-1α expression and mitochondrial biogenesis, thereby reducing mitochondrial mass and respiratory capacity (67). In chronically stimulated T-cells (*e.g*., in the tumor or persistent antigen context), researchers found a progressive loss of PGC-1α and other metabolic regulators, resulting in small, fragmented, and dysfunctional mitochondria in “exhausted” T-cells (68). Analogously, in surgical patients, persistent inflammation and catecholamine exposure can drive T-cells into a dysfunctional state where their mitochondria cannot meet energy demands. High levels of cortisol and catecholamines postoperatively also induce lymphocyte mitochondrial dysfunction by inhibiting glycolysis and diverting metabolism (this is part of the so-called CARS – compensatory anti-inflammatory response syndrome). Clinically, it’s been observed that mitogen-induced ATP production in patient T-cells is lower after major surgery and correlates with nosocomial infection risk (69, 70). Additionally, surgery often involves anesthesia drugs that can directly or indirectly affect mitochondria – *e.g.*, propofol in high doses can inhibit complex I of the electron transport chain (contributing to Propofol infusion syndrome in rare cases); volatile anesthetics can depolarize mitochondria mildly (which in heart preconditioning is protective, but in immune cells could DAMPsen function) (28).

##### 2.1.3.4 Altered macrophage polarization (M1/M2 balance)

Macrophages are plastic and can shift phenotypes depending on cues – classically activated M1 are pro-inflammatory and microbicidal, whereas alternatively activated M2 are anti-inflammatory and involved in repair (**Figure 2**). Mitochondrial metabolism is a key determinant of this polarization: M1 macrophages rely on glycolysis and have broken TCA cycles (as described), whereas M2 macrophages rely on intact OXPHOS and fatty acid oxidation, with high mitochondrial spare respiratory capacity (45). After surgery, the systemic milieu (high HMGB1, IL-6, *etc.*) tends to drive monocytes/macrophages toward an M1 state. If their mitochondria are concurrently damaged, they may get “stuck” in a chronic M1-like state because insufficient OXPHOS prevents the transition to M2. An elegant study demonstrated that inhibition of OXPHOS with respiratory chain poisons prevented M1→M2 repolarization even when M2 stimuli (IL-4) were given. Conversely, restoring mitochondrial function (*e.g.*, providing oxaloacetate or boosting ETC flux) pushed macrophages towards an M2 profile (21). In obesity research, it was shown that mitochondrial dysfunction in adipose tissue macrophages kept them in an M1 state and contributed to chronic inflammation and insulin resistance (71). By analogy, during the perioperative period, if patient macrophages cannot effectively perform mitochondrial respiration, they may perpetuate inflammation and also fail to perform their healing roles. Moreover, surgery often causes a surge in myeloid-derived suppressor cells (MDSCs) – many of which are metabolically reprogrammed monocytes/neutrophils that suppress T-cells and promote tumor growth (in cancer patients). These MDSCs exhibit high glycolysis and low OXPHOS, and strategies to target their metabolism (*e.g.*, inhibit glycolysis) are being investigated as a way to prevent postoperative cancer metastasis (72). Thus, immune cell mitochondrial function is directly tied to the balance of pro- *vs.* anti-inflammatory responses after surgery.

##### 2.1.3.5 Lymphocyte mitochondrial apoptosis and autophagy

In surgical patients, particularly those who experience major blood loss or shock, lymphocyte counts often drop (lymphopenia). Apoptosis of T-cells and B-cells can be triggered by extrinsic factors (*e.g.*, Fas ligand, cortisol) but also by intrinsic mitochondrial pathways (*e.g.*, BAX/BAK-mediated cytochrome c release) due to cellular stress. Mitochondrial membrane permeabilization is a point-of-no-return in apoptosis. IR injury in secondary lymphoid organs and high oxidative stress can tip lymphocytes into apoptosis, thereby reducing adaptive immune capacity postoperatively (73). At the same time, autophagy (including mitophagy) in T-cells might be induced by surgical stress as a survival mechanism. Some immunomodulatory effects of anesthetic drugs are mediated by autophagy; for example, dexmedetomidine has been shown to enhance mitochondrial autophagy in microglia, reducing neuroinflammation and cognitive impairment in a mouse model of anesthetic exposure (74–76). Fine-tuning of mitochondrial turnover in immune cells can therefore influence their survival and function under perioperative stress. Excessive autophagy might lead to immune cell deletions (immunosuppression), whereas defective autophagy leads to accumulation of dysfunctional mitochondria (inflammation). Therapies that modulate autophagy – like rapamycin (which induces autophagy *via* mTOR inhibition) – have shown in rodents to mitigate postoperative cognitive dysfunction by promoting clearance of damaged mitochondria in the brain (77).

In summary, the perioperative period is a perfect storm for immune cell mitochondria: abrupt environmental changes (oxygen, temperature, blood flow), massive inflammatory and neuroendocrine signals, and high energy demands all converge on immune cell metabolic pathways. The result can be mitochondrial dysfunction manifesting as: diminished ATP generation, excess ROS, altered substrate use (glucose *vs.* fatty acids), and activation of cell death or senescence programs. These changes underlie phenomena like “surgical immunoparalysis” (where patients become immunosuppressed after the initial inflammatory hit) and also contribute to prolonged inflammation that delays recovery.

From a mechanistic perspective, key takeaways include (**Table 1**):

**Table 1:** Mitochondrial alterations in immune cells during the perioperative period.

Immune cell type	Baseline mitochondrial metabolism	Perioperative alterations	Functional consequences	Key references
Neutrophils	Predominantly glycolytic ATP production with minimal reliance on oxidative phosphorylation (few mitochondria, mainly for signaling).	Surgical stress triggers mitochondrial dysfunction: loss of membrane potential and surge in mitochondrial ROS, leading to accelerated neutrophil apoptosis in the immediate postoperative period. Major trauma/surgery also induces release of mitochondrial DNA, fueling neutrophil extracellular trap (NET) formation *via* TLR9.	Early postoperative neutropenia and impaired neutrophil function (diminished pathogen clearance). Excess NETs and ROS can damage tissues and promote complications (*e.g.*, organ injury or tumor spread).	(81–83)
Monocytes/Macrophages	High metabolic plasticity. Resting/”M0” monocytes use both glycolysis and OXPHOS; classically activated M1 macrophages favor glycolysis and a broken TCA cycle, while anti-inflammatory M2 macrophages rely on intact TCA cycle and β-oxidation.	Perioperative stress and anesthesia reprogram myeloid metabolism. Peripheral monocytes show increased glycolysis after major surgery. Surgical anesthesia can skew macrophages toward an anti-inflammatory, M2-like state (elevated IL-10, TGF-β, CD163^+^ cells) indicating postoperative immunosuppressive polarization. Non-classical monocytes may remain functionally impaired for ≥48h post-surgery.	Shifts toward M2 polarization and glycolysis are associated with postoperative immune suppression – reduced bactericidal activity and anti-tumor surveillance. This may increase infection risk and cancer metastasis. Conversely, excessive M1 response can heighten inflammatory tissue damage.	(83–86)
T lymphocytes (CD4^+^ & CD8^+^)	Naïve and memory T cells primarily utilize oxidative phosphorylation and fatty-acid oxidation (high mitochondrial mass in memory cells), whereas activated effector T cells switch to aerobic glycolysis to meet energetic demands. T cell subsets have distinct metabolic programs (*e.g.*, memory T cells boast greater spare respiratory capacity than effector cells).	Major surgery causes transient lymphopenia due to mitochondrial oxidative stress and apoptosis in T cells. At ~24 h post-op, peripheral lymphocytes exhibit a collapse of Δψm and glutathione depletion alongside elevated ROS. Lymphocyte counts and mitoprotection typically recover by 3–4 days post-op, though regulatory T cells may remain dysregulated for >48 h.	Acute loss of T cells (postsurgical lymphocytopenia) leads to weakened adaptive immunity. Impaired T helper and cytotoxic responses (reduced IL-2, IFN-γ) increase susceptibility to infections and potentially allow tumor escape. Mitochondrial dysfunction in T cells can also promote exhaustion. Recovery of T cell function over days helps restore immune homeostasis, but prolonged T_reg alterations could affect inflammation *vs.* tolerance balance.	(86–88)

Understanding these mechanisms sets the stage for interventions. If we can prevent mtDAMPs release or support mitochondrial function in immune cells, we may attenuate the entire downstream cascade of perioperative organ injury. The next sections will explore the array of therapeutic strategies aimed at precisely this goal – treating mitochondria as a lever to recalibrate the immune response in surgical patients.

##### 2.1.3.6 Mitochondrial health dictates immune cell phenotype

Robust mitochondrial function promotes balanced immunity (*e.g.*, enables macrophages to heal, T-cells to form memory), whereas mitochondrial dysfunction skews toward either hyper-inflammation (if DAMPs are released) or hypo-inflammation (if energy failure leads to cell exhaustion) (71, 78).

##### 2.1.3.7 Surgery recapitulates metabolic syndromes

There are parallels between the immune metabolic changes in surgery and those in other conditions – for instance, the “cytokine storm” and subsequent “immune exhaustion” in severe COVID-19 or sepsis have similar roots in mtDAMPs release and metabolic collapse. This cross-talk suggests that therapies tested in sepsis (like IL-1 blockers, metabolic cofactor supplementation) could be repurposed in high-risk surgical patients.

##### 2.1.3.8 Patient factors (age, comorbidity) matter

An older patient or one with diabetes may already have baseline mitochondrial dysfunction and altered immunometabolism (*e.g.*, chronic low-grade inflammation, reduced mitochondrial content in immune cells) (79, 80). Surgery on this background might push immune cells over the edge. This is why elderly patients have higher rates of postoperative delirium, infections, and organ dysfunction – their immune mitochondria are less resilient. It also implies potential benefit of prehabilitation strategies (like exercise or nutritional supplements pre-surgery) that can “boost” mitochondrial function prior to the insult (72).

#### 2.1.4 Acute T cell dysfunction and perioperative immunosuppression

T cell dysfunction is common after major surgery and critical illness, but it is not necessarily synonymous with the terminal exhaustion described in cancer and chronic viral infection. Classical exhaustion is typically driven by prolonged antigenic stimulation and is characterized by progressive loss of effector function, sustained expression of multiple inhibitory receptors (including programmed cell death protein 1, PD-1), and—critically—a stable exhaustion-associated epigenetic landscape that constrains reinvigoration even after checkpoint blockade (89). In contrast, perioperative “immune paralysis” often arises within days, tracks with systemic inflammation and metabolic stress, and may be partially reversible as homeostasis is restored, resembling the transient immunosuppressive state described in sepsis rather than chronic antigen exposure (6).

A key unresolved question is whether perioperative immune dysfunction engages the core transcriptional circuitry of exhaustion. TOX/TOX2 and NR4A1–3 function as central regulators that cooperate to establish and maintain an exhaustion program through chromatin remodeling and epigenetic stabilization (90, 91). This “programmatic” component distinguishes exhaustion from short-lived activation-associated PD-1 upregulation. Currently, direct evidence that early postoperative T cell suppression consistently exhibits TOX/NR4A-dominant modules is limited; available observations are more consistent with bioenergetic insufficiency and transient metabolic constraint, which can phenocopy dysfunction without implying epigenetic commitment (67).

Mechanistically, mitochondrial stress provides a plausible bridge between these states. ROS accumulation, loss of membrane potential, and impaired oxidative phosphorylation can limit T cell proliferation and cytokine production, and may also potentiate inhibitory receptor signaling when stress is sustained (23). We propose that the duration and persistence of mitochondrial injury—potentially reinforced by ongoing mtDAMP exposure during prolonged critical illness—may determine whether postoperative dysfunction remains reversible or transitions toward an exhaustion-like state accompanied by TOX/NR4A activation and durable immunosuppression (92).

Therapeutically, this distinction has practical implications. Metabolically constrained, potentially reversible dysfunction may be more amenable to strategies that restore mitochondrial fitness (e.g., redox modulation) and support lymphocyte recovery (e.g., IL-7/IL-15), whereas exhaustion-like states may require checkpoint modulation in combination with approaches that target upstream mitochondrial stress and the TOX/NR4A–epigenetic axis. Identifying bedside biomarkers that separate transient dysfunction from programmatic exhaustion remains a critical challenge.

### 2.2 Ischemia-reperfusion injury: a central mechanism of mitochondrial dysfunction in perioperative immune cells

Among the various triggers of mitochondrial dysfunction, IR injury stands out as a central mechanistic axis in perioperative settings. IR injury represents a fundamental pathophysiological process that directly precipitates mitochondrial dysfunction in immune cells during the perioperative period. During high-risk surgical procedures—including cardiac surgery with cardiopulmonary bypass, organ transplantation, major vascular surgery with aortic cross-clamping, and trauma resuscitation—tissues inevitably experience episodes of ischemia followed by reperfusion. This IR cascade initiates a characteristic sequence of metabolic and oxidative perturbations within mitochondria: succinate accumulation during ischemia, reverse electron transport-driven ROS generation upon reperfusion, calcium overload, opening of the mPTP, and ultimately, release of mtDAMPs. These molecular events not only compromise immune cell bioenergetics and effector functions but also propagate sterile inflammation throughout the systemic circulation. This section delineates the IR-mitochondria axis as a central mechanistic framework for understanding perioperative immune dysregulation and its downstream consequences for multi-organ protection.

#### 2.2.1 The ischemia-reperfusion cascade: from metabolic stress to microvascular dysfunction

The IR cascade follows a well-defined temporal sequence initiated by oxygen deprivation. During ischemia, the absence of oxygen as the terminal electron acceptor halts oxidative phosphorylation and forces cells into anaerobic glycolysis. Consequently, succinate dehydrogenase (SDH) reverses its catalytic direction, reducing fumarate to succinate using electrons from reduced ubiquinone, leading to substantial succinate accumulation (up to 10-fold increases) within minutes. This metabolic shift also impairs mitochondrial complex I function and reduces mitochondrial membrane potential (ΔΨm), priming the organelle for subsequent injury (93). Upon reperfusion and reoxygenation, the accumulated succinate is rapidly re-oxidized by SDH, driving extensive reverse electron transport (RET) at mitochondrial complex I. This RET event generates a burst of superoxide and downstream ROS, establishing the primary oxidative insult of reperfusion injury. The ROS burst precipitates mitochondrial Ca^2+^ overload through activation of the mitochondrial calcium uniporter (MCU), further depolarizing ΔΨm and triggering opening of the mPTP. Notably, mPTP opening represents a critical decision point in cell fate: transient, low-conductance openings serve physiological roles in Ca^2+^ homeostasis, whereas sustained, high-conductance opening exceeding the “point of no return” inevitably leads to mitochondrial swelling, outer membrane rupture, and cell death via apoptosis or necrosis (94).

Concurrently, IR injury profoundly compromises microvascular integrity. Ischemia-induced endothelial cell activation, expression of adhesion molecules (P-selectin, ICAM-1), and reperfusion-associated ROS production promote leukocyte-endothelial adhesion, capillary plugging, and endothelial barrier dysfunction. The resulting “no-reflow” phenomenon—wherein tissue perfusion remains inadequate despite macrovascular restoration—exacerbates hypoxia and creates a self-amplifying cycle of inflammation, mitochondrial damage, and microcirculatory failure (95).

#### 2.2.2 mtDAMPs release and SIRS-like systemic inflammation

A pivotal consequence of IR-induced mitochondrial damage is the amplification of mtDAMPs release into the extracellular space and systemic circulation. These evolutionarily conserved molecules—including mtDNA, N-formyl peptides, cardiolipin, and mitochondrial transcription factor A (TFAM)—bear bacterial molecular signatures due to the endosymbiotic origin of mitochondria. When released from disrupted cells following mPTP opening and mitochondrial swelling, mtDAMPs function as potent endogenous danger signals that activate innate immune pathways identical to those triggered by microbial pathogens. Circulating mtDAMPs activate polymorphonuclear neutrophils (PMNs) and monocytes through formyl peptide receptor-1 (FPR1), Toll-like receptor 9 (TLR9), and the NLRP3 inflammasome, inducing Ca^2+^ flux, mitogen-activated protein (MAP) kinase phosphorylation, neutrophil migration, degranulation, and cytokine release. In trauma and hemorrhagic shock models, elevated circulating mtDAMPs levels correlate with injury severity and drive a SIRS that clinically mimics sepsis—a phenomenon termed “sterile sepsis” or “autosepsis.” Notably, experimental administration of purified mtDAMPs is sufficient to recapitulate neutrophil-mediated organ injury and hemodynamic collapse, establishing a direct causal link between IR-induced mitochondrial disruption and SIRS-like systemic inflammation (16).

The IR-mtDAMPs axis creates a feed-forward amplification loop: IR injury causes mitochondrial damage and mtDAMPs release; mtDAMPs activate neutrophils and monocytes, which infiltrate tissues and release additional ROS and proteases, causing further mitochondrial damage and cell death; this secondary damage releases more mtDAMPs, perpetuating the inflammatory cascade and amplifying the SIRS response beyond the initial ischemic insult.

#### 2.2.3 Multi-organ injury and postoperative complications

The IR-mtDAMPs-SIRS axis provides a mechanistic framework for understanding multi-organ dysfunction following high-risk surgery. Specific examples include:

Pulmonary injury: Following abdominal aortic surgery or liver transplantation, gut IR and hepatic mtDAMPs release drive acute lung injury via the gut-lung axis. Circulating mtDAMPs activate pulmonary neutrophils and endothelial cells, causing increased vascular permeability, alveolar edema, and acute respiratory distress syndrome (ARDS)—a major cause of postoperative morbidity and prolonged mechanical ventilation (96).

Renal injury: Aortic cross-clamping and cardiopulmonary bypass create renal ischemia, leading to tubular mitochondrial damage, mtDAMPs release, and neutrophil-mediated tubular injury. This manifests as AKI, requiring renal replacement therapy in severe cases and significantly increasing mortality risk (97).

Cardiac injury: Myocardial IR during cardiac surgery generates substantial mtDAMPs release, contributing to post-cardiotomy syndrome (fever, leukocytosis, organ dysfunction) and low cardiac output syndrome. Ventricular arrhythmias arising from reperfusion injury represent a leading cause of postoperative hemodynamic instability (98).

Neurological injury: Cerebral and spinal cord IR during aortic surgery with circulatory arrest causes delayed paraplegia and cognitive dysfunction. mtDAMPs-mediated neuroinflammation and microvascular dysfunction exacerbate ischemic neuronal injury, with limited therapeutic options currently available (16).

#### 2.2.4 Implications for therapeutic strategies

The IR-mtDAMPs-organ injury axis provides a mechanistic framework for perioperative organ protection. Current research is actively investigating interventions targeting specific nodes of this cascade, including succinate dehydrogenase inhibition, cyclophilin D modulation, TLR9 antagonism, and mitochondrial transplantation (99).

### 2.3 Mitochondrial redox signaling and permeability transition in perioperative immune cells

The IR-mtDAMPs axis described above does not operate in isolation. While ROS have received considerable attention in perioperative stress responses, mitochondrial redox biology encompasses a broader repertoire of reactive species, ion fluxes, and membrane dynamics that collectively determine immune cell viability and function. This section examines how RNS, Ca^2+^ dysregulation, and the mPTP intersect with ROS to shape perioperative immune pathology. NO and peroxynitrite are produced by iNOS in activated macrophages and neutrophils during IR injury. These RNS modify respiratory chain complexes I and IV through tyrosine nitration, disrupt inner membrane integrity via cardiolipin oxidation, and directly damage mtDNA—effects that amplify rather than merely parallel ROS-mediated injury (100, 101). The resultant respiratory dysfunction exacerbates electron leak and further ROS generation, establishing a self-amplifying cycle. Ca^2+^ homeostasis represents another critical vulnerability. Oxidative and nitrative stress compromise plasma membrane and SR/ER Ca^2+^-ATPase activity, raising cytosolic Ca^2+^ and driving mitochondrial uptake through MCU. Moderate matrix Ca^2+^ stimulates dehydrogenases and supports ATP production; however, when buffering capacity is exceeded—particularly under sustained oxidative stress—the mPTP opens. This channel, formed from F_1_F_0_-ATP synthase and regulated by cyclophilin D, functions as a molecular decision point for cell fate (102). The mPTP exhibits distinct gating modes with profound functional consequences. Transient, low-conductance openings permit matrix Ca^2+^ efflux and volume regulation without compromising outer membrane integrity; these represent reversible, adaptive responses. Sustained, high-conductance opening exceeds a “safe return point,” triggering matrix swelling, outer membrane rupture, and regulated cell death. Pharmacological inhibition of cyclophilin D is only effective before this threshold is crossed, defining a narrow therapeutic window (94).

Together, these observations establish that perioperative mitochondrial dysfunction arises from the integrated action of ROS, RNS, Ca^2+^ overload, and mPTP dynamics—not ROS alone. This mechanistic complexity explains the consistent failure of nonspecific antioxidant strategies in clinical trials and supports the development of targeted interventions: mitochondria-targeted antioxidants that accumulate selectively in the matrix; peroxynitrite decomposition catalysts; MCU inhibitors that prevent Ca^2+^ overload; and cyclophilin D ligands that modulate mPTP gating. Preconditioning protocols may further confer protection by raising the threshold for mPTP opening, effectively shifting the safe return point and expanding the intervention window (103, 104).

### 2.4 Extreme inflammation: MAS and CRS

In severe cases, uncontrolled mtDAMP release may push SIRS into macrophage activation syndrome or cytokine storm—life-threatening states with explosive IL-1β, IL-6, IL-18 and TNF-α elevation, plus hemodynamic collapse. Macrophage activation syndrome (MAS) features uncontrolled macrophage activation with hemophagocytosis; Cytokine release syndrome (CRS) involves massive T cell cytokine release. Both represent the IR-mtDAMP axis running unchecked, where persistent mitochondrial damage overwhelms regulatory mechanisms. These syndromes are uncommon in routine surgery but illustrate what happens when mitochondrial inflammation escapes control, justifying early intervention in high-risk patients (105, 106).

## 3 Therapeutic strategies targeting immune cell mitochondria

**Figure 5:**
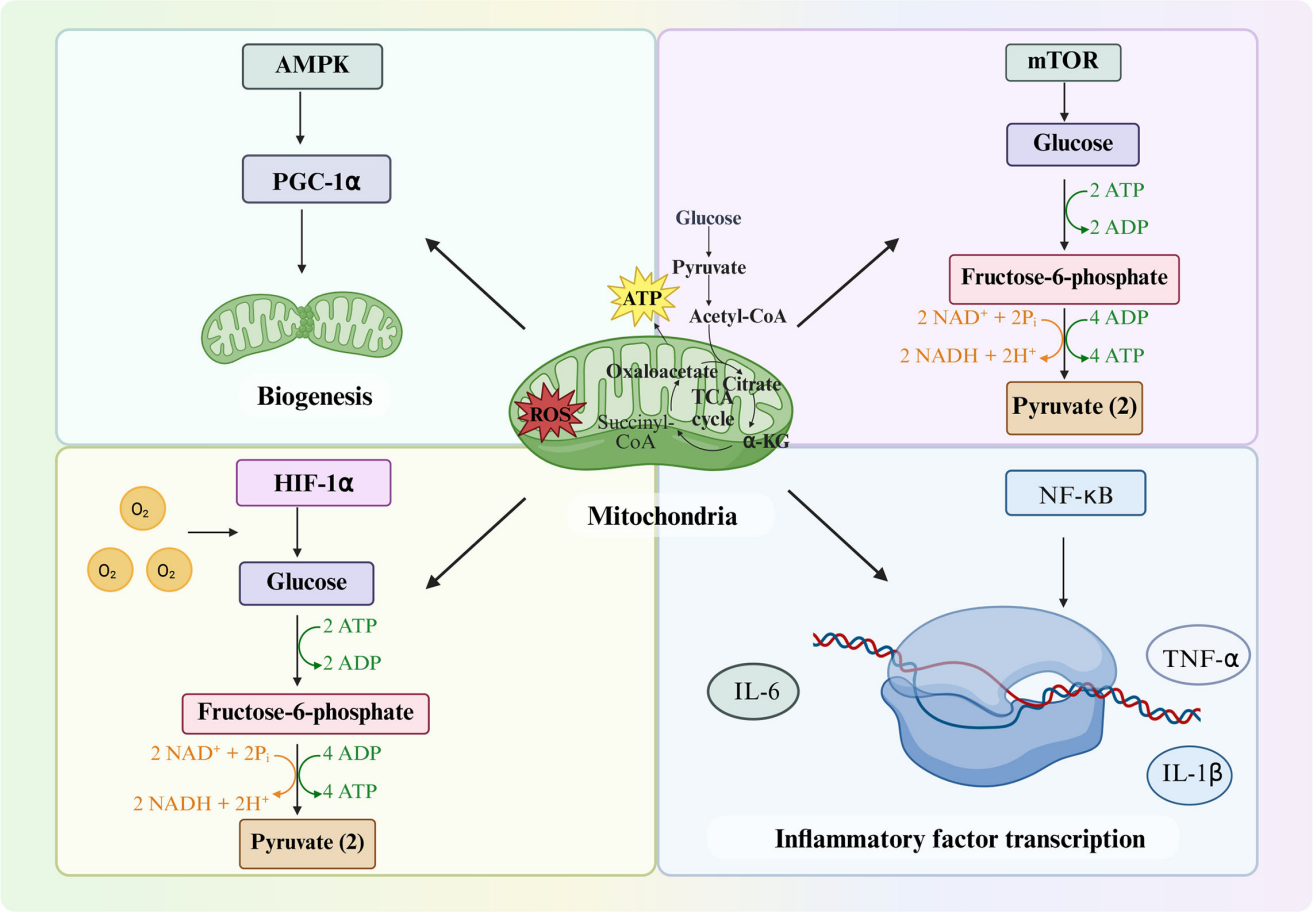
Mitochondrial metabolism in different immune cell types.

Advancements in our understanding of immunometabolism and mitochondrial signaling have opened new avenues for therapeutic intervention (**Figure 5**). Rather than broadly suppressing the immune system (as with high-dose steroids, which have mixed results in surgical patients), these emerging strategies aim to fine-tune immune function by modulating mitochondrial behavior – essentially treating the root causes of dysregulation rather than the symptoms. We can categorize these strategies into three groups: pharmacological agents, biologic therapies, and genetic/cellular approaches. Many therapies overlap categories (*e.g.*, a peptide could be seen as both a drug and a biologic), but the classification is useful for organizational purposes (**Table 2**).

**Table 2:** Therapeutic strategies targeting immune cell mitochondria.

Strategy	Agents/examples	Mechanism of action	Preclinical/clinical evidence	References
Antioxidant therapy	Mitochondria-targeted antioxidants (*e.g.*, MitoQ, MitoTEMPO, SS-31 peptide); systemic antioxidants (N-acetylcysteine, vitamin C, melatonin, coenzyme Q10).	Scavenge mitochondrial ROS and stabilize mitochondrial membrane potential, thereby reducing oxidative damage to immune cells. Mitigate release of mtDAMPs and prevent activation of cell death pathways.	*Preclinical:* MitoQ lowered mtROS, preserved Δψm, and suppressed pro-inflammatory cytokines in cell and rodent sepsis models. *Clinical:* IV NAC in cardiac surgery patients significantly reduced postoperative AKI and atrial arrhythmias. Postoperative melatonin (5–10 mg) in CABG patients improved cardiac function (↑ EF) and antioxidant capacity, with decreased inflammatory markers.	(157–159)
Metabolic modulators	Agents adjusting immune cell fuel usage: *e.g.*, Dichloroacetate (DCA); AMP-kinase activators (metformin); glutamine supplementation; ketogenic substrates (β-hydroxybutyrate).	Reprogram immune cell metabolism to optimize mitochondrial function. For example, DCA activates pyruvate dehydrogenase, shifting metabolism from glycolysis to OXPHOS (reducing lactate accumulation). Metformin and β-hydroxybutyrate enhance AMPK/SIRT3 pathways, improving mitochondrial efficiency and biogenesis.	*Preclinical:* DCA reversed tumor-induced T cell dysfunction – reducing immunosuppressive arginase-1 and boosting IFN-γ^+^ CD8^+^ T cells and NK cells by alleviating lactic acidosis. In mouse sepsis, ketogenic interventions and metformin have shown reduced inflammatory cytokines and preserved ATP levels (various studies). *Clinical:* Pilot studies are exploring DCA for lactic acidosis in surgical ICU patients, and metformin is being repurposed for perioperative organ protection (data emerging).	(127)
Gene therapy (mito-targeted)	Mitochondrial gene augmentation or editing: *e.g.*, mtDNA editing (mitoZFN or TALEN to remove mutations); gene delivery of mitochondrial enzymes or regulators (TFAM, catalase); *ex vivo* T cell modification (PGC-1α overexpression).	Directly correct or enhance mitochondrial function in immune cells *via* genetic means. Strategies include removing deleterious mtDNA mutations or upregulating biogenesis regulators. Enhanced expression of PGC-1α (master regulator of mitochondrial biogenesis) increases mitochondrial content and respiratory capacity in immune cells.	*Preclinical:* Enforced PGC-1α expression in CD8^+^ T cells elevated their mitochondrial oxidative capacity and spare respiratory reserve, promoting formation of long-lived memory T cells with superior antitumor function in mice. Mice receiving T cells transduced with PGC-1α showed improved tumor control. Ongoing research in models of sepsis and aging demonstrates that viral delivery of mitochondrial antioxidant enzymes (like catalase) can reduce immune cell apoptosis. *Clinical:* Gene therapy for immune cell mitochondria is still in experimental stages; early trials for inherited mitochondrial diseases use nucleus-coded gene delivery to complement mtDNA defects.	(88, 160)
Mitochondrial transplantation	Isolation of healthy mitochondria (often autologous) and transfusion into target tissues or cells. Techniques include direct injection or carrier-mediated delivery of mitochondria.	Donor mitochondria are taken up by recipient cells, where they integrate or signal to improve bioenergetics. Transplanted mitochondria can replace or rejuvenate damaged mitochondria in immune or tissue cells, restoring ATP production and reducing cell injury. They may also trigger mitophagy of the recipient’s unhealthy mitochondria.	*Preclinical:* In a murine intracerebral hemorrhage model, IV delivery of magnetically guided mitochondria improved microglial mitochondrial function, shifting them to a less pro-inflammatory state and aiding neurological recovery. *Clinical:* Autologous mitochondrial transplantation has been piloted in children undergoing cardiac surgery. In a case series (16 pediatric patients), direct injection of the child’s own mitochondria into post-ischemic myocardium led to 80% ECMO weaning success (*vs.* ~40% historically), with improved ventricular function and no adverse immune reaction. Trials are underway in adult heart transplant optimization (DCD hearts) using mitochondrial transfer.	(161)
Mitophagy/biogenesis enhancers	Pharmacological inducers of mitophagy or mitochondrial biogenesis: *e.g.*, Urolithin A (a gut metabolite that activates mitophagy), NAD^+^ precursors (nicotinamide riboside), sirtuin activators (resveratrol), and PPAR agonists. Also, lifestyle interventions (exercise, intermittent fasting) which upregulate PGC-1α.	These strategies boost the turnover of dysfunctional mitochondria and/or increase the formation of new mitochondria. Enhanced mitophagy removes damaged mitochondria in immune cells, reducing inflammation (since fewer mtDAMPs released), while increased biogenesis improves the cells’ energy resilience.	*Preclinical:* Urolithin A has been shown to promote mitophagy and restore mitochondrial function in macrophages and microglia, thereby attenuating NLRP3 inflammasome-mediated inflammation. It also induced formation of memory T cells with greater mitochondrial fitness in animal tumor models. *Clinical:* A recent trial in older adults found Urolithin A to be safe and to upregulate mitochondrial gene expression in CD8^+^ T cells (suggesting enhanced mitophagy). Early-phase studies of NAD^+^ boosters indicate improved immune cell mitochondrial respiration in sepsis patients (ongoing). Endurance exercise prehabilitation before surgery can increase leukocyte PGC-1α levels and might improve postoperative immune mitochondrial health (investigational).	(162, 163)

### 3.1 Pharmacological agents (small-molecule drugs and nutrients)

#### 3.1.1 Mitochondria-targeted antioxidants

One of the earliest approaches to protecting mitochondria has been using antioxidants that specifically accumulate in mitochondria. Traditional antioxidants (like vitamin C, N-acetylcysteine) have had limited success in complex inflammatory conditions, partly because they do not reach mitochondria in sufficient concentrations. Mitochondria-targeted antioxidants such as MitoQ (mitoquinone) and SS-31 (elamipretide) were designed to overcome this. MitoQ is a ubiquinone (coenzyme Q10) analog attached to a lipophilic triphenylphosphonium cation, which drives its uptake into the negatively-charged mitochondrial matrix. SS-31 is a cell-permeable peptide that inserts into the inner mitochondrial membrane and stabilizes cardiolipin, reducing electron leak and lipid peroxidation. In animal models of cardiac arrest and hemorrhagic shock, MitoQ and SS-31 have been shown to reduce markers of oxidative damage, preserve ATP levels, and attenuate end-organ injury. For example, SS-31 given to rodents during resuscitation from hemorrhagic shock improved survival and reduced lung injury by lowering mitochondrial ROS in immune cells (107). Mechanistically, SS-31 is capable of preventing the opening of the mPTP and sustaining mitochondrial electron transport; thereby preventing the burst of ROS and mtDNA that would otherwise trigger inflammation (107). In the context of hepatic ischemia-reperfusion injury associated with liver surgery, SS-31 can inhibit M1 polarization of macrophages, reduce pro-inflammatory cytokines, and thereby alleviate liver injury by scavenging ROS and regulating STAT1/STAT3 signaling (108). MitoQ has also been studied in sepsis to investigate whether it can improve patients’ prognosis by inhibiting the inflammatory cascade (109, 110). These agents exhibit excellent overall safety profiles, with only a few mild adverse reactions observed. Take MitoQ as an example: it may cause mild hypotension, and mechanistically, this is presumably related to the drug regulating mitochondrial function in smooth muscle, which in turn induces vasodilation. Even melatonin, a hormone with strong antioxidant properties that accumulates in mitochondria, has been shown to reduce organ injury in various IR models and has been trialed in liver surgery context as a hepatoprotective (with some positive results in reducing transaminase release) (111). Overall, mitochondrial antioxidants aim to break the cycle of ROS → mtDNA release → inflammation, and thus serve as upstream protectors.

#### 3.1.2 Inflammasome and DAMPs inhibitors

Since NLRP3 inflammasome activation and mtDNA-TLR9 are key drivers, pharmacological inhibition of these can be beneficial. NLRP3 inhibitors like 16673-34-0, an intermediate substrate free of the cyclohexylurea moiety involved in insulin release, have shown efficacy in preclinical models of myocardial IR (reduced infarct size by ~30% and improved ejection fraction) (112). While not yet tested in surgical patients, there is a related drug Dapansutrile (OLT1177) – an orally active NLRP3 inhibitor – which is in trials for gout and heart failure. It could feasibly be repurposed perioperatively (*e.g.*, high-risk cardiac surgery) to prevent IL-1 mediated injury (113, 114). Neutralizing IL-1β or IL-18 (the inflammasome products) is another approach: Anakinra (IL-1 receptor antagonist) has been used off-label in systemic inflammation settings and is attractive for short-term use around surgery to DAMPsen the IL-1 surge (115). Continuous intravenous infusion of anakinra can effectively control severe and life-threatening cytokine storms caused by diseases such as MAS; additionally, its application in other cytokine storm syndromes, including CRS associated with CAR-T cell therapy, is worthy of exploration (116). Targeting TLR9 – the receptor for mtDNA – is also promising. CpG ODN 2088 is a TLR9 antagonist oligonucleotide which, in a pig CPB study, reduced plasma IL-6 by ~90% and improved kidney function (34). While not yet in clinical use, similar compounds could be given just prior to surgery to prevent the mtDNA from over-activating immunity. Additionally, DNase I infusion to degrade extracellular DNA (both nuclear and mito) has shown benefit in trauma models by reducing inflammation (117). There is an ongoing trial of intravenous DNase in trauma patients for that reason. In the context of surgery, prophylactic DNase might reduce postoperative SIRS (though could have potential side effect of impairing host defense against infection, so careful balance needed).

#### 3.1.3 Metabolic modulators

##### 3.1.3.1 Classical metabolic modulators

These drugs adjust the substrate utilization or metabolic pathways of immune cells. For example, Metformin, an AMPK activator and complex I inhibitor, has been suggested to confer organ protection *via* multiple mechanisms: it reduces hyperglycemia (high sugars can worsen mitochondrial injury), activates AMPK which stimulates antioxidant defenses and mitophagy, and skew metabolism away from pure glycolysis. Perioperative insulin resistance and stress hyperglycemia are common after major surgery and are associated with oxidative stress and organ injury; thus, agents that simultaneously improve glycemic control and mitochondrial quality control may be particularly relevant in perioperative settings (1, 4, 64). Retrospective studies found that diabetic patients on metformin had lower incidence of AKI after cardiac surgery (118). Beyond glycemic effects, ischemia–reperfusion injury is strongly linked to mitochondrial ROS generation at reperfusion (including succinate-associated mechanisms), providing a rationale for exploring perioperative “mitochondria-targeted” metabolic modulation in IR-prone procedures such as cardiopulmonary bypass (93, 95). Metformin also reduces NLRP3 inflammasome activation in macrophages by promoting autophagic clearance of damaged mitochondria (119, 120). At the signaling level, AMPK antagonizes mTORC1, a central node controlling the glycolysis–oxidative metabolism balance; in macrophages this axis can constrain inflammatory glycolysis and inflammasome activity, whereas in T cells it supports mitochondrial fitness and persistence—processes that appear perturbed after major surgery (19, 22).

Beta-hydroxybutyrate (BHB), a ketone body, is another interesting example – BHB directly inhibits the NLRP3 inflammasome by preventing K^+^ efflux and possibly *via* activation of the hydroxycarboxylic acid receptor 2 (HCA2) on immune cells (121, 122). In mice, raising BHB levels (either by fasting or administering exogenous ketones) protected against kidney IR injury and reduced IL-1β levels (123). This renal IR evidence is directly pertinent to perioperative AKI risk, particularly in settings featuring transient hypoperfusion or clamp-related ischemia (1). Clinically, ketogenic diets or supplements could theoretically be used preoperatively to precondition patients, although feasibility and patient acceptance can be issues.

Sodium nitrite and Sildenafil are being evaluated for their mitochondria-protective effects *via* enhancing NO signaling and blood flow, which can reduce reperfusion injury (nitrite can inhibit mitochondrial complex I in a reversible manner to reduce ROS burst on reperfusion). In a lung ischemia–reperfusion model, nitrite attenuated mitochondrial impairment and vascular permeability, supporting the concept that NO-dependent mitochondrial modulation can mitigate reperfusion-associated injury (124).

Cyclosporine A, traditionally an immunosuppressant, at low doses blocks the mPTP. It was tested in a large myocardial infarction trial (CIRCUS trial) but did not significantly reduce infarct size in humans; however, in smaller cardiac surgery studies, cyclosporine reduced some markers of myocardial injury (125). This is consistent with the established role of mPTP opening early during reperfusion as an integration point for Ca^2+^ overload and oxidative stress (102, 103).

The variability in results suggests that timing (giving it before the ischemia *vs.* at reperfusion) is crucial. Melatonin, mentioned earlier, in human trials of abdominal aortic aneurysm repair reduced oxidative stress markers and appeared to lessen intestinal injury (126). It acts as both a direct free radical scavenger and through melatonin receptors that strengthen mitochondrial function (melatonin increases the activity of electron transport chain complexes and upregulates antioxidant enzymes). Because melatonin is very safe, some centers have started giving it (usually 10–20 mg orally at night) for a few days around big surgeries as a potential neuro- and cardioprotective adjunct. Additional clinical data in CABG patients suggest postoperative melatonin reduces oxidative stress and inflammatory markers and is associated with improved cardiac function, further supporting its perioperative translational relevance (127). Given that major surgery can induce T-cell immunometabolic paralysis linked to mitochondrial damage, mitochondria-supportive interventions may also be mechanistically relevant to postoperative immunosuppression (60).

##### 3.1.3.2 Anesthetic agents as immunometabolic modulators

The drugs we use routinely in anesthesia can be leveraged as “immunometabolic” modulators. Volatile anesthetics (*e.g.*, sevoflurane) are well known to cause ischemic preconditioning – part of this effect is *via* mild uncoupling of mitochondria and activation of cytoprotective kinases (like PKC-ϵ) that ultimately reduce mPTP opening on reperfusion (28, 128, 129). Clinically, volatile anesthetics have been associated with lower troponin release and less myocardial ischemia than propofol in some cardiac surgery trials (130). Opioid analgesics like morphine activate μ-opioid receptors which can trigger pro-survival signaling in heart and also reduce sympathetic tone (lowering metabolic demand). Morphine also inhibits NLRP3 inflammasomes in macrophages (*via* κ-opioid receptors on immune cells) in some studies (131). Dexmedetomidine, a sedative, deserves special mention: Dex acts on α2-adrenergic receptors to reduce catecholamine release and has been repeatedly shown to attenuate inflammation and organ injury in surgical and ICU patients (132, 133). At the cellular level, Dexmedetomidine helps preserve mitochondrial ultrastructure and function. For instance, in septic rat models, Dex prevented mitochondrial morphology disruption and maintained ATP production in vital organs (134). It also upregulated heme oxygenase-1 (HO-1) and other antioxidant pathways, likely through HIF-1α activation, thereby reducing oxidative stress (135). These cytoprotective effects have also been validated in clinical practice. Dexmedetomidine used in cardiac surgery patients during the perioperative period, it can not only reduce the incidence of AKI and postoperative atrial fibrillation but also shorten the length of stay in the ICU (136). A meta-analysis, further confirmed that administering an appropriate dose of dexmedetomidine can significantly decrease the levels of key pro-inflammatory factors such as interleukin-6 (IL-6) and tumor necrosis factor (TNF), and improve the functional indices of vital organs like the heart and kidneys (132). Thus, based on a clear mechanism of action and sufficient experimental and clinical evidence, the strategy of using dexmedetomidine as an anesthetic adjuvant for organ protection in high-risk surgeries is increasingly becoming a routine clinical choice.

#### 3.1.4 Other metabolic therapies

*Vitamin B3 (niacinamide) and NAD^+^ precursors* are being explored to boost cellular NAD^+^ levels, which decline during ischemia and with age. NAD^+^ is required for sirtuin enzymes that promote mitochondrial biogenesis and DNA repair. Trials of nicotinamide riboside in critical illness are starting. Iron chelators like *Deferoxamine* can prevent iron-catalyzed ROS (Fenton chemistry) and have been shown to reduce neuroinflammation and cognitive deficits after surgery in rodent models by mitigating ferroptosis (iron-dependent cell death) in neurons (137). Sodium bicarbonate or tris-hydroxymethyl aminomethane (THAM) can be used to correct acidosis which, aside from improving hemodynamics, may relieve the inhibition of metabolic enzymes by low pH. There is evidence that controlling acidosis reduces mitochondrial dysfunction in shock. Finally, therapeutic hypothermia (mild cooling to 33–35 °C) is a physical intervention that slows metabolic rate and has mitochondrial-protective effects (it reduces mitochondrial Ca^2+^ overload and ROS production). It’s standard after cardiac arrest and is being tested in intraoperative settings like deep hypothermic circulatory arrest. However, hypothermia in general surgery can be harmful (impairs coagulation, *etc.*), so the key is targeted organ cooling (like cold perfusion for transplants or topical cooling of the heart/brain).

In sum, a host of pharmacologic agents – from novel small molecules to repurposed anesthetic drugs – are being utilized to stabilize mitochondria, reduce DAMPs release, and optimize immune cell metabolism during the perioperative period. Often, combinations may be synergistic (*e.g.*, using Dexmedetomidine for its sympathetic attenuation plus a mitochondrial antioxidant for direct ROS scavenging). The optimal agent may differ by surgical context (for example, an IL-1 blocker might be more relevant in a context with high inflammasome activation like transplantation, whereas a TLR9 blocker might be ideal for cases with major tissue injury). Ongoing and future trials will clarify which of these pharmacological interventions can translate into reduced organ injury and improved patient outcomes.

### 3.2 Biologic therapies (peptides, cytokine modulators, and cell-derived vesicles)

#### 3.2.1 Mitochondria-targeted peptides and proteins

We already mentioned SS-31 (elamipretide) which is technically a peptide (so it can be considered a biologic). Another peptide in development is Szeto-Schiller peptide 20 (SS-20), which, unlike SS-31, is not positively charged but still binds cardiolipin and was designed to reduce apoptosis (138). In addition, the mitochondria-derived peptide MOTS-c is a notable mitochondria-targeted biological agent. Encoded by mitochondrial 12S rRNA, this 16-amino-acid peptide exists in human tissues (*e.g.*, liver, muscle) and the circulatory system. Studies show it has multiple functions: regulating insulin sensitivity and metabolic homeostasis, preventing age- and high-fat diet-induced insulin resistance/obesity in mice, protecting the heart in cardiovascular diseases *via* relevant pathways, and reducing inflammation when intraperitoneally administered. Despite clinical application challenges, introducing it into safe probiotics *via* synthetic biology for precise expression offers a new application direction (139). A related study employed a rodent model to assess the therapeutic efficacy of a novel fusion protein (Exscien I-III) that targets mitochondrial DNA. The study found that this fusion protein could regulate mitochondria-related functions, reduce the apoptosis of isolated rat islets cultured with pro-inflammatory cytokines, enhance their viability, and maintain islet function. In the donor brain death (BD) rat model, the fusion protein attenuated the inflammatory response induced by brain death and significantly enhanced the insulin secretion function of transplanted islets in response to glucose stimulation both *in vitro* and *in vivo* (140). Overall, mitochondria-targeted peptides and proteins exhibit functions such as regulating metabolism, protecting the heart, reducing inflammation, and improving islet function. Although some of them face challenges in clinical application, they can be introduced into safe vectors through synthetic biology techniques to achieve precise expression, which provides a new path for expanding application directions.

#### 3.2.2 Cytokine and immune-modulatory biologics

Instead of targeting the mitochondria directly, some biologics aim to modulate the immune consequences of mitochondrial dysfunction. For example, IL-6 blockade (Tocilizumab) could curb the deleterious systemic effects of IL-6 released upon mtDNA-TLR9 activation. Tocilizumab is used in CAR-T cell therapy to stop cytokine storms and has been trialed in cardiac surgery to see if it reduces post-op atrial fibrillation (since IL-6 is implicated). Preliminary results show it lowers CRP and fibrinogen but effect on AF is still under investigation. TNF-α blockers (like Etanercept) have been less studied in surgical context but could hypothetically reduce some mitochondrial damage, as TNF contributes to mPTP opening and mitochondrial apoptosis pathways. However, blocking TNF carries infection risks, so likely not prophylactic.

#### 3.2.3 IL-10 or IL-22 therapy

These are anti-inflammatory cytokines. IL-10 is the body’s natural brake on inflammation, enhancing macrophage resolution phase and improving mitochondrial function (it promotes oxidative metabolism in macrophages). There have been experimental attempts to give IL-10 or IL-10 mimetics perioperatively to high-risk patients (*e.g.*, major liver resection) to reduce complications. IL-22 is another cytokine that helps regeneration of gut and liver and can mitigate mitochondrial damage in those tissues. A designer fusion of IL-22 to an antibody (to extend half-life) is in trials for preventing acute graft-versus-host disease after bone marrow transplant, and conceptually could be used for gut protection in big surgeries like aortic aneurysm repair to reduce intestinal permeability and subsequent sepsis.

#### 3.2.4 Biologics reducing neutrophil extracellular traps

NETs can spill mtDNA and histones, fueling inflammation. DNase I, as mentioned, is a biologic enzyme that can digest NETs. Another target is PAD4 (peptidyl arginine deiminase 4), the enzyme required for NET formation; Cl-amidine is a PAD4 inhibitor being explored in sepsis models. By reducing NETs, one reduces sources of mitochondrial (and nuclear) DAMPs in circulation (141).

#### 3.2.5 Extracellular vesicles and exosomes

Mesenchymal stem cells (MSCs) have been investigated for their role in organ protection in sepsis and acute respiratory distress syndrome (ARDS), primarily due to their ability to secrete beneficial exosomes that can transfer mitochondria or mitochondrial components to other cells (142). In ARDS model studies, it has been found that MSCs “donate” mitochondria to macrophages; after acquiring these mitochondria, the macrophages exhibit a significant increase in oxidative phosphorylation efficiency, along with a marked enhancement in their ability to phagocytose pathogens and clear damaged substances (143). Some companies are looking into **“mitovesicles”** – basically exosomes loaded with functional mitochondria or antioxidants – that could be given IV to patients to distribute protective signals. This is in very early stages but holds intriguing possibility for cell-free mitochondrial therapy.

#### 3.2.6 Mitochondrial transplantation (cell-based biologic therapy)

A groundbreaking new approach is direct mitochondrial transplantation (MTx). This involves isolating mitochondria from a donor source (often the patient’s own tissue, like a small piece of skeletal muscle), purifying them, and then infusing or injecting them into the target organ or bloodstream. Remarkably, studies have shown that exogenous mitochondria can be taken up by cells and remain functional (27, 144). The initial success was reported by McCully et al., where autologous mitochondria injected into ischemic rabbit hearts led to improved post-ischemic function (98). This was translated into a compassionate use case in a pediatric patient undergoing heart transplantation – mitochondrial transplantation into the patient’s heart (which had suffered a prolonged ischemic time) was associated with recovery of cardiac function (145). Following that, a phase I trial in children undergoing heart surgery showed the procedure to be feasible and safe (146). The proposed mechanism is two-fold: transplanted mitochondria can directly supply ATP to energy-starved cells and can also modulate immune cells by reducing DAMPs release and apoptosis. In a mouse cardiac arrest model, Hayashida et al. found that injecting mitochondria IV after resuscitation improved survival and organ function; interestingly, they noted reductions in systemic inflammatory cytokines and changes in immune cell gene expression favoring resolution of inflammation (147). This suggests that MTx not only provides energy but also sends anti-inflammatory signals, possibly through restoring the metabolic balance in immune cells. Challenges for MTx include scaling up mitochondria for adult patients, timing (it likely needs to be done very soon after ischemic insult for maximal benefit), and ensuring the mitochondria reach target organs (local delivery *vs.* systemic). If autologous source is used, it requires taking a biopsy quickly, isolating mitochondria (~30 minutes process), and administering them, which may fit in some surgical settings (like before removing cross-clamp in heart surgery). Alternatively, allogenic mitochondria (from cell lines or unrelated donors) are being explored – if they don’t cause adverse immune reactions, they could be an “off-the-shelf” product for emergencies.

#### 3.2.7 Engineered immune cells for improved metabolism

While more of a cell therapy than classical biologic, it’s worth noting efforts in modifying immune cells to enhance their mitochondrial function. For instance, researchers have experimented with CAR-T cells overexpressing PGC-1α (the transcriptional coactivator that drives mitochondrial biogenesis). These CAR-T cells showed increased mitochondrial content, better OXPHOS, and were more resistant to the tumor microenvironment (less exhausted) (78, 148). Such metabolically “fit” T-cells could potentially be used after cancer surgery or for immunotherapy in sepsis. Similarly, memory T-cells enriched in infusions (with high spare respiratory capacity) could be given to patients who are severely immunosuppressed after big surgery to prevent infections. These concepts are still experimental, but the science is moving toward tailoring immune cell metabolism *ex vivo* and then using those cells as a therapy. One example entering trials is “*ex vivo* cytokine-treated monocytes” – essentially training a patient’s monocytes to be more anti-inflammatory and better at phagocytosis by culturing them with IL-4/IL-10, then infusing them back (this approach is being tested in sepsis under the name “GM-CSF tuned monocytes”). Part of their efficacy might come from corrected mitochondrial function (IL-4 would push them towards an M2 phenotype with robust mitochondria).

#### 3.2.8 Nanoparticle delivery systems

Nanomedicine is aiding biologic strategies by enabling targeted delivery.

For instance, macrophage-derived nanovesicles (NVs) loaded with ursodeoxycholic acid (UDCA) and modified with triphenylphosphine (TPP) (designated as UDCA-NVs-TPP) can specifically accumulate in damaged mitochondria, alleviate oxidative stress, and increase ATP production by 42.62% to improve cellular function (149). Nanoliposomes dual-modified with the brain injury site-targeting peptide CAQK and TPP can deliver 4-octyl itaconate (4-OI) to the mitochondria of cells in the brain, inhibiting the activation of the NLRP3 inflammasome and the opening of the mPTP to alleviate neuroinflammatory responses (150). Additionally, human serum albumin nanoparticles camouflaged with red blood cell (RBC) membranes and modified with the neuron-targeting molecule T807 and TPP (T807/TPP-RBC-NPs) possess excellent biocompatibility and long circulation properties; they can cross the blood-brain barrier to target neuronal mitochondria, and when loaded with curcumin, reduce mitochondrial oxidative stress and inhibit neuronal death (151). A lutein nanoassembly (lutein@DTPP) constructed based on DSPE-thioketal-PEG2K enables myocardial targeting and ROS-responsive drug release, improving mitochondrial function by regulating the MDM2-NDUFS1 axis and inhibiting ferroptosis in myocardial cells during myocardial ischemia-reperfusion injury (152). Beyond these, NO-releasing nanoparticles have also been tested in septic mice: they improved microcirculation and modulated inflammation by acting on immune cell mitochondria—low-dose NO gently inhibits mitochondrial respiration and reduces ROS production (153). These nanoparticle systems collectively provide effective solutions for targeted improvement of mitochondrial dysfunction and alleviation of the pathological processes of related diseases, demonstrating broad application potential.

In summary, biologic approaches provide more target specificity and can harness or mimic the body’s own regulatory mechanisms. Whether by supplying healthy mitochondria, blocking key inflammatory cytokines, or reprogramming immune cells, these strategies complement the pharmacologic ones. In fact, combinations are conceivable: *e.g.*, giving a mitochondrial transplant along with an IL-1 inhibitor to both restore energetics and block residual inflammation. The biologics often act at a higher level of the cascade (*e.g.*, IL-1 blocker doesn’t stop mtDNA release but stops its effect), whereas some drugs act at the mitochondrial level (like MitoQ stops the ROS that would lead to IL-1 production). As research progresses, the hope is that some of these biologic modalities will enter clinical practice, especially in high-stakes surgeries (transplantation, cardiac surgery, major trauma) where current supportive care is not always sufficient to prevent organ injury.

### 3.3 Genetic and cellular engineering approaches

The field of genetic and cellular therapies overlaps with biologics but warrants separate discussion because it involves longer-term modifications or specialized cell products that could fundamentally alter immune cell behavior perioperatively.

#### 3.3.1 Enhancing mitochondrial biogenesis *via* gene therapy

One concept is to upregulate key mitochondrial regulators in patient’s own cells. For instance, delivering genes like PGC-1α, TFAM (mitochondrial transcription factor A), or NRF2 (antioxidant response master regulator) to immune cells or organs. Viral vectors (like adeno-associated viruses, AAV) can be used to carry these genes. In the hepatic IR injury model, AAV-mediated overexpression of PGC-1α protects the liver by activating PPARα/PPARγ and regulating ROS (154). TFAM is a core molecule for mtDNA maintenance and transcription. In transgenic mouse experiments, TFAM overexpression not only increased the mtDNA copy number in mouse hearts by approximately 2-fold but also, under volume overload (VO) conditions, inhibited the production of mitochondrial reactive oxygen species (mitoROS), reduced the activity and abnormal upregulation of MMP-2/9, ultimately alleviating eccentric myocardial hypertrophy, improving cardiac function, and causing no damage to mitochondrial enzyme activity (155). However, such gene therapy in humans is limited by timing—it takes several days to weeks to fully take effect, making it more suitable for preconditioning in elective surgeries. An alternative *ex vivo* modification approach is also available: for instance, patients’ monocytes can be isolated one week before surgery, and the intracellular TFAM expression level can be upregulated *via* lentiviral transfection to enhance mtDNA maintenance capacity. After reinfusion before surgery, this is theoretically expected to improve monocytes’ resistance to surgical stress and efficiency in clearing tissue debris. In summary, enhancing mitochondrial biogenesis through gene therapy can be achieved either by delivering genes like PGC-1α and TFAM *via* AAV, or by regulating the expression of relevant factors in monocytes through preoperative *ex vivo* lentiviral transfection—both contributing to preconditioning for elective surgeries.

#### 3.3.2 RNA-based therapy

Instead of delivering genes, one could deliver siRNAs or antisense oligonucleotides to modulate gene expression. For example, an siRNA against mitochondrial fission protein Drp1 could potentially reduce excessive mitochondrial fragmentation in immune cells (excess fission is seen in stress and leads to dysfunction). Or antisense oligonucleotides to mtDNA could perhaps bind and prevent mtDNA from activating TLR9 (though delivering to endosomes in immune cells is a challenge). With advances in lipid nanoparticles for RNA (as seen with mRNA vaccines), it’s plausible to have mRNA therapeutics that encode protective mitochondrial proteins delivered transiently.

#### 3.3.3 CAR-T and CAR-NK with metabolic enhancements

We discussed how CAR-T cells engineered for better metabolism show improved function in cancer. In the perioperative realm, one could imagine using such modified immune cells to either fight residual cancer after resection or to combat postoperative infections when the native immune cells are dysfunctional. For example, CAR-NK cells engineered to resist hypoxia (perhaps *via* HIF stabilizers or extra copies of glycolytic enzymes) could be used in liver transplant patients to clear infection in an oxygen-poor environment. Some preclinical work has looked at T-cells overexpressing antioxidant enzymes (like catalase) to see if they persist better in oxidatively stressed environments. Indeed, T-cells modified to overexpress catalase had improved survival in tumor models (156). The translation would be immune cells that could be given to patients undergoing surgery as a supportive immune boost that is resistant to the oxidative/inflammatory surgical milieu.

#### 3.3.4 Stem cell therapies

Beyond MSCs mentioned prior, induced pluripotent stem cell (iPSC)-derived immune cells could be made with defined mitochondrial characteristics (for example, selecting clones with high mitochondrial mass or with certain mitochondrial DNA haplotypes known for efficient respiration). These could serve as universal donor cells to modulate immune response. An area of interest is iPSC-derived regulatory T-cells or tolerogenic dendritic cells for transplant surgeries – they could promote immune regulation and possibly carry better mitochondria if pre-conditioned in culture (there’s evidence that culture conditions can improve mitochondrial health of cells).

#### 3.3.5 Microbiome and metabolite therapy

It’s tangential but notable that the gut microbiome can release metabolites (like short-chain fatty acids) that influence host mitochondria and immunity. Prebiotic or probiotic strategies might indirectly modulate immune mitochondria. For example, butyrate from gut microbes is an HDAC inhibitor that can improve regulatory T-cell mitochondrial function and anti-inflammatory activity. Fecal transplants or certain diets might thus be considered a form of “biotherapy” altering the host’s metabolic-inflammatory axis prior to surgery.

#### 3.3.6 Precision medicine and biomarker-guided therapy

A key aspect of future directions is combining these therapies with patient selection using biomarkers. If a patient is identified (*via* high circulating mtDNA or low monocyte HLA-DR, *etc.*) as being in a state of impending immunopathology, then more aggressive mitochondrial-targeted interventions could be applied. Conversely, patients without those signals might avoid unnecessary interventions. This is more of a strategy than a therapy but it relies on integrating biomarker knowledge (like measuring mtDNA kinetics, mitochondrial respiration in blood cells *ex vivo*, *etc.*) – which can be seen as a tool emerging from our mechanistic insights.

Many of the genetic/cellular strategies are still in nascent phases, but they represent the bleeding edge of therapy – moving beyond mitigating damage to actively reprogramming immune cells to be resilient. These could potentially induce long-lasting changes (for example, enhancing a patient’s immune system for weeks to get through a high-risk postoperative period).

**Figure 6:**
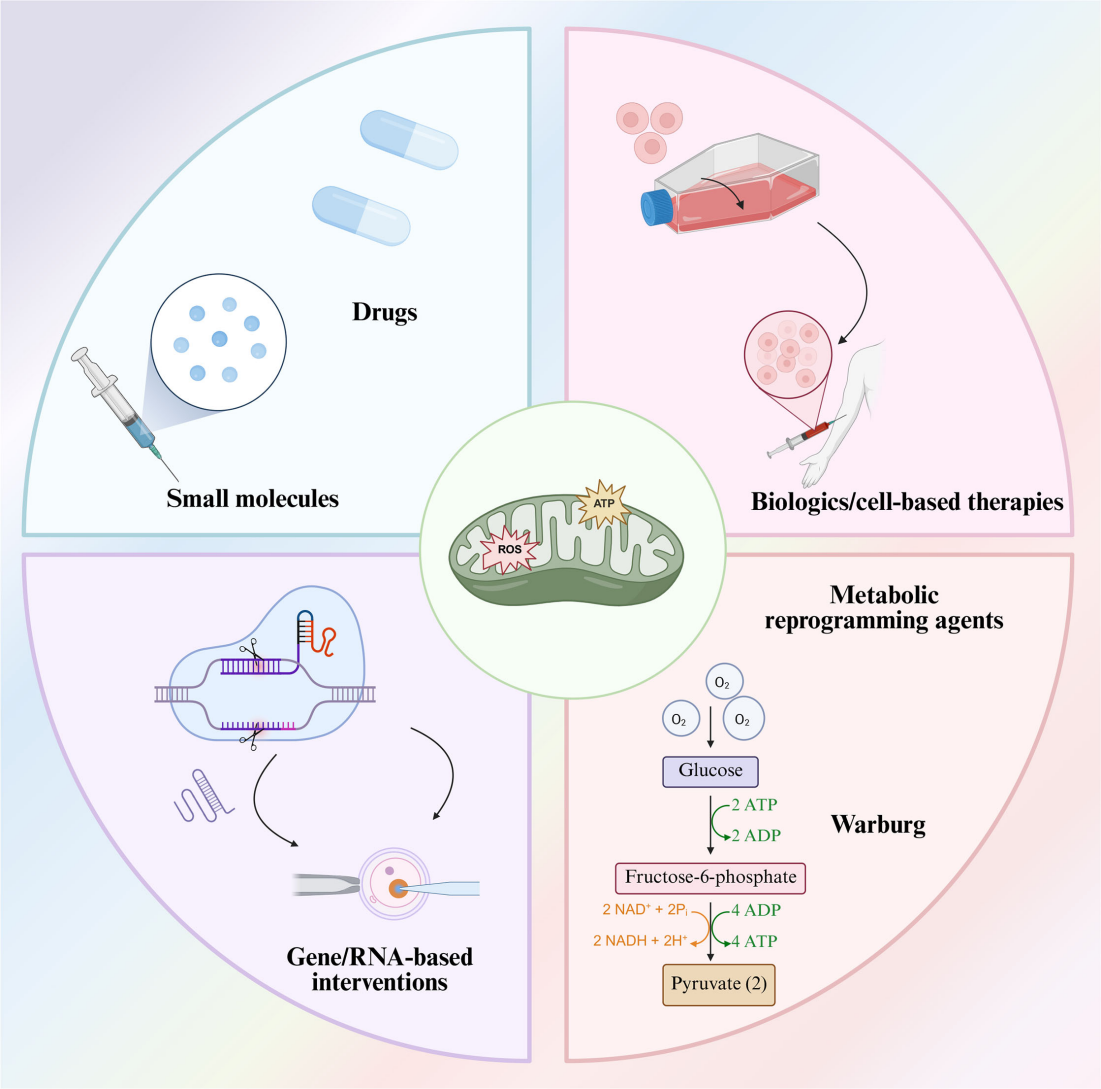
Therapeutic strategies targeting immune cell mitochondria.

**Figure 7:**
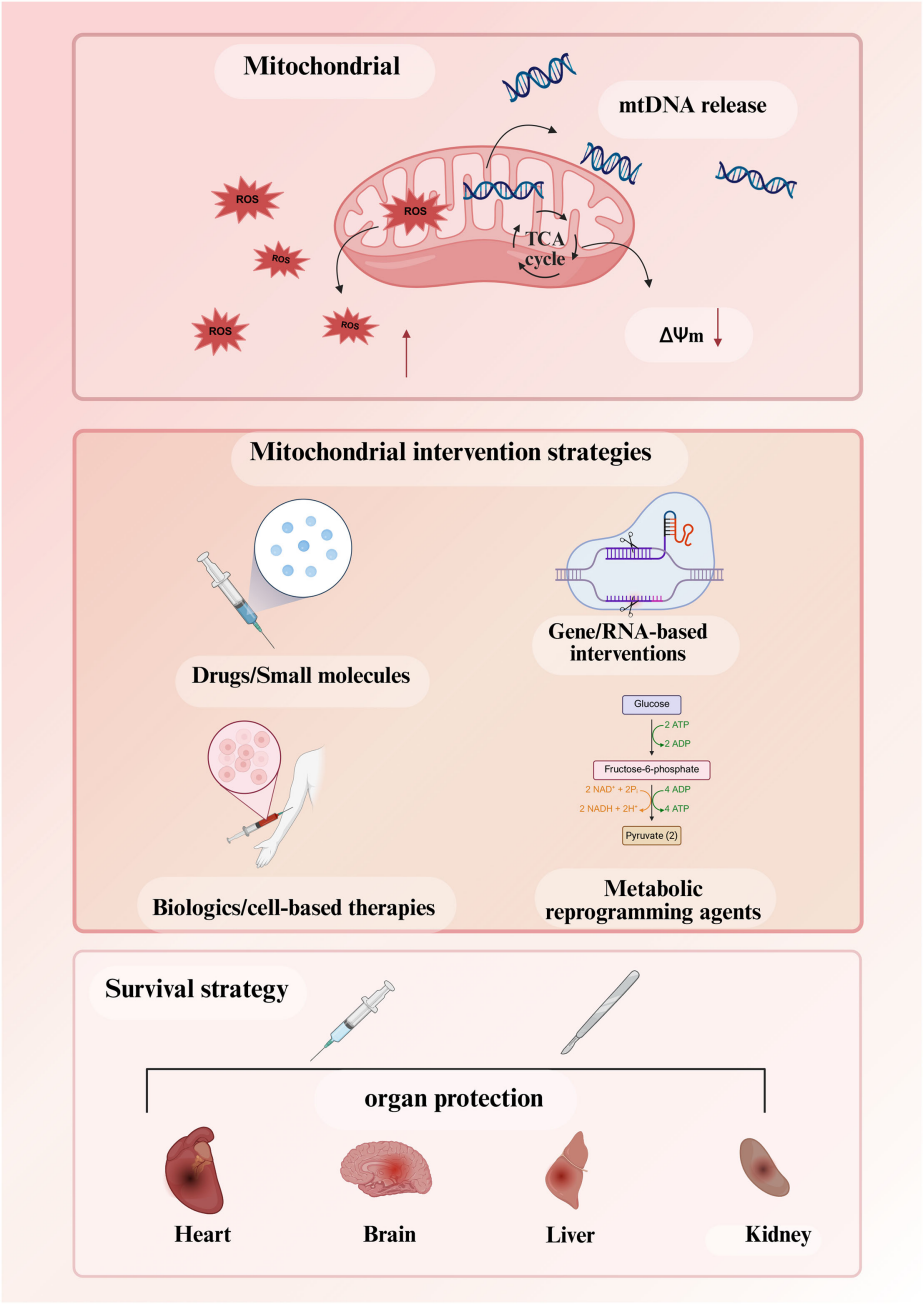
From mechanism to clinical translation.

In conclusion, a spectrum of interventions is being developed to modulate perioperative immunity via mitochondrial pathways. **Figure 2** illustrates how these interventions map onto the immunometabolic pathways: e.g., scavenging mtDNA, inhibiting inflammasomes, boosting mitophagy, providing healthy mitochondria, and improving immune cell metabolism at various points in the cascade. **Figure 6** details these therapeutic strategies targeting immune cell mitochondria. **Figure 7** diagrams how such interventions have been applied in different organs (heart, brain, kidney) and what outcomes have been observed or expected, bridging mechanism to clinical translation.

Before discussing organ-specific evidence, it’s worth noting that many of these therapies have shown success in preclinical models but not all have been validated in large clinical trials. The complexity of human perioperative pathophysiology means combination therapies might be needed (for instance, an antioxidant plus an IL-1 blocker might work better than either alone, addressing multiple feedback loops). Additionally, timing (prophylactic *vs.* after injury onset) is critical – preventing mitochondrial dysfunction is easier than reversing it after it has triggered cell death pathways. Therefore, a guiding principle emerging is that early intervention in the perioperative timeline (pre- or intra-operative) yields the best chance to alter the trajectory of organ injury.

With the mechanistic and therapeutic groundwork laid, we will now delve into clinical evidence from major surgical domains – examining how these concepts manifest in real-world patient data and trials, and where gaps remain between bench and bedside.

## 4 Clinical evidence in heart, brain, liver, and kidney surgeries

Translating mitochondrial immunomodulation into improved clinical outcomes requires corroborating evidence from surgical patient studies. A growing body of such evidence demonstrates that perioperative mitochondrial dysfunction—manifested as elevated circulating mtDNA, impaired respiratory function, or altered mitochondrial dynamics—predicts adverse postoperative outcomes including SIRS, organ dysfunction, and mortality (**Table 3**). In this section, we review key findings from different surgical contexts: cardiac surgery (often involving global ischemia and extracorporeal circulation), neurosurgery (with focus on brain inflammation and cognitive outcomes), and abdominal surgeries involving liver and kidney ischemia (such as transplant or major resections). These organ systems exemplify how immune cell mitochondrial dysfunction can influence surgical recovery and how interventions targeting these pathways have fared clinically (**Figure 7**).

**Table 3:** Clinical studies linking perioperative mitochondrial dysfunction to postoperative outcomes.

Surgical type	Sample size	Mitochondrial dysfunction marker	Postoperative outcome	Main finding	References
Mixed ICU (including postoperative patients)	361	Circulating mtDNA	Mortality	Plasma mtDNA levels independently associated with ICU mortality, with predictive value superior to APACHE II score	(96)
Cardiac surgery	50	Circulating mtDNA	SIRS, organ dysfunction	Postoperative mtDNA peak correlated with SIRS severity and AKI occurrence	(16)
Trauma (including surgical patients)	143	Circulating mtDNA	Mortality, MODS	mtDNA levels correlated with ISS score; predicted MODS and death	(164)
Sepsis (including postoperative sepsis)	115	Skeletal muscle mitochondrial ATP production, complex I activity	Mortality	Mitochondrial dysfunction (reduced ATP production) independently associated with sepsis mortality; metabolic resuscitation concept	(165)
Cardiac surgery	80	Circulating mtDNA, mitochondrial respiratory function	Acute kidney injury	Postoperative mtDNA elevation preceded and predicted AKI development	
Cardiac surgery (canine model)	16	Mitochondrial transplantation	Myocardial function	Injection of isolated mitochondria during early reperfusion reduced infarct size and improved post-ischemic functional recovery	(98)
Cardiac ischemia-reperfusion (murine)	—	Succinate accumulation, reverse electron transport (RET), mitochondrial ROS	Reperfusion injury	Ischemic succinate accumulation drives RET at complex I upon reperfusion, generating ROS burst; metabolic signature of IR injury	(93)

### 4.1 Cardiac surgery (heart and systemic organ protection)

Cardiac surgeries like coronary artery bypass grafting (CABG) or valve replacement commonly utilize cardiopulmonary bypass (CPB), which triggers a significant systemic inflammatory response due to blood contact with artificial surfaces, ischemia-reperfusion of the heart, and global hypothermia/rewarming (166, 167). The result is often a SIRS in the immediate postoperative period. Clinically, this can manifest as vasodilatory shock, pulmonary dysfunction, coagulopathy, arrhythmias (like atrial fibrillation), and AKI (168). The severity of the inflammatory response correlates with complications – *e.g.*, patients with higher post-CPB IL-6 or IL-8 have more postoperative atrial fibrillation and longer ICU stays. As discussed, mtDAMPs are major instigators of this inflammation in cardiac surgery.

#### 4.1.1 Evidence of mtDAMPs release

Multiple studies have measured cell-free mitochondrial DNA (cf-mtDNA) in cardiac surgery patients. One study found that cf-mtDNA levels rose dramatically upon aortic cross-clamp release and peaked in the early postoperative hours (33). Patients who developed postoperative organ dysfunction (like AKI or lung injury) had significantly higher peak mtDNA levels than those who didn’t (52, 169). A 2025 pilot study in surgical aortic valve replacement patients reported that cf-mtDNA increased ~16-fold during CPB and that those with higher mtDNA had more postoperative infection and longer hospital stays (33). Another study noted that TLR9 and NLRP3 gene expression in circulating leukocytes increased after CPB in proportion to cf-mtDNA, linking the DAMPs to receptor activation (51). Importantly, as evidence of causation, animal models like the one by Naase et al. showed blocking TLR9 during CPB (with a TLR9 antagonist) significantly reduced IL-6 release and also improved cardiac contractility and reduced troponin levels post-CPB (34). Similarly, adding an antioxidant (sulforaphane) during CPB in pigs reduced circulating mtDNA by 75% and blunted IL-6 (170), demonstrating that preventing mitochondrial oxidative damage can reduce DAMPs release.

#### 4.1.2 Inflammation and organ injury in heart surgery

Cardiac surgery patients commonly experience myocardial stunning (temporary post-ischemic cardiac dysfunction). Inflammatory cytokines like TNF and IL-6, whose release is partly driven by mtDAMPs, depress myocardial contractility (171). These cytokines plus activated complement also increase endothelial permeability, contributing to tissue edema in the heart and lungs. This is one reason for difficulty weaning from bypass and need for inotropes/vasopressors (a hyperdynamic circulation with low SVR is characteristic of post-CPB SIRS) (33). If severe, this inflammatory vasoplegia can progress to a vasoplegic shock syndrome. Organs like the kidney are very susceptible – cardiac surgery-associated AKI occurs in up to 30% of patients after complex surgery. Research implicates neutrophil activation and mtDNA-TLR9 pathways in CPB-AKI: in patients who developed AKI, higher urinary mtDNA and IL-8 were found, indicating neutrophil influx and possibly local mtDAMPs from kidney ischemia (172). On histology, these patients show acute tubular necrosis with neutrophil trapping. Trials of interventions like statins (pleiotropic metabolic modulators) and remote ischemic preconditioning (RIPC) have yielded mixed results on AKI, but notably RIPC – which triggers brief limb ischemia to induce systemic protective signals – has been shown in some studies to reduce plasma mtDNA and IL-1β after CPB (173).

#### 4.1.3 Interventions and outcomes

Dexmedetomidine: Many cardiac surgery centers now use Dexmedetomidine infusion intraoperatively or postoperatively for sedation. A large meta-analysis of cardiac surgery patients found that Dex use was associated with a reduction in incidence of postoperative atrial fibrillation (POAF) and a reduction in duration of mechanical ventilation (136). Mechanistic smaller studies showed that Dex-treated patients had lower peak IL-6, lower troponin I release (indicating less myocardial injury) (174), and less AKI (136). In a study of patients undergoing cardiac valve replacement surgery with cardiopulmonary bypass (CPB), patients were randomly assigned to the dexmedetomidine (Dex) group or the placebo group. The Dex group received a loading dose before anesthesia induction, followed by a maintenance dose until the end of the surgery, while the placebo group was given normal saline. Results showed that the Dex group had lower levels of serum urea nitrogen (BUN), creatinine (Cr), and neutrophil gelatinase-associated lipocalin (NGAL) at certain postoperative time points, higher intraoperative urine output, and a significantly reduced incidence of AKI, indicating that this drug can improve renal function and reduce the risk of kidney injury (175). These organ-protective effects are attributed to mitigation of hyperinflammation and maintenance of tissue oxygen utilization – consistent with Dex’s mitochondrial benefits.
Remote Ischemic Preconditioning (RIPC): In the field of cardiac surgery, remote ischemic preconditioning (RIPC) triggers a systemic protective effect through “transient limb ischemia-reperfusion”. Its core mechanism lies in mitochondrial regulation, which specifically involves reducing mitochondrial damage, regulating apoptosis and oxidative stress-related pathways, and transmitting protective signals *via* humoral factors (176, 177). Studies have shown that RIPC can reduce myocardial infarction size and troponin levels by regulating calcium channel subunits, and it can also inhibit the NOX4-ROS signaling pathway to alleviate renal mitochondrial dysfunction, thereby lowering the risk of postoperative renal injury (178, 179). However, large-scale multicenter trials such as ERICCA and RIPHeart have not demonstrated its ability to improve major outcomes like 30-day postoperative mortality; only subgroup analyses suggest that patients with prolonged intraoperative ischemia may benefit. Overall, RIPC is a potential “mitochondria-targeted” strategy for protecting the heart and kidneys from ischemia-reperfusion injury, and may be more valuable for patients with prolonged ischemia. Nevertheless, there are controversies in current research results, and direct evidence of mitochondrial protection (*e.g.*, association with mtDNA) is insufficient, requiring more research breakthroughs to promote its standardized clinical application.
Statins: Statins (HMG-CoA reductase inhibitors) have antioxidant and anti-inflammatory effects. Some randomized trials of high-dose atorvastatin preoperatively did not show a reduction in AKI across all patients, but one trial noted fewer AKI cases in statin-naïve patients given rosuvastatin before cardiac surgery (controversial due to other trials being neutral). In terms of the mechanism of action, statins can act on the calcium channel subunit Cacna2d3 to reduce calpain 1-mediated myocardial mitochondrial apoptosis, thereby exerting a protective effect against myocardial ischemia-reperfusion injury. This process also indirectly reflects the regulation of mitochondrial function by statins, and since mitochondrial function is closely related to inflammatory responses and the like, it can provide a basis for statins to exert anti-inflammatory and other effects (180). Observational data suggest patients on chronic statins have lower inflammatory cytokines after CPB and shorter ICU stays (181).
Methylprednisolone: Some practices give steroids (like a one-time dose of methylprednisolone) before CPB to blunt the inflammatory response. The SIRS of high-dose methylprednisolone did not reduce mortality or major complications, although it did reduce postoperative IL-6 levels significantly (182). However, it also increased infection risk in some patients. Steroids likely stabilize lysosomal and mitochondrial membranes (reducing mtDNA release) but their broad immunosuppression is a double-edged sword. Hence, the move is toward more targeted immunomodulation (like IL-6 or IL-1 blockade) rather than global steroid use.
Mitochondrial-targeted trials: In clinical research on regulating mitochondrial function, mitochondrial - targeted peptides and small - molecule drugs are important approaches. As a typical mitochondrial - targeted peptide, SS-31 (elamipretide) can stabilize cardiolipin in the inner mitochondrial membrane, reduce electron leakage to inhibit ROS, inhibit the opening of mPTP to reduce the release of mtDNA and cell apoptosis, protect mitochondria and exert anti - inflammatory effects, and is effective on immune cells and parenchymal cells such as cardiomyocytes. Researchers have conducted a Phase 2 clinical trial of this drug in patients with cardiovascular and renal diseases. The results showed that elamipretide can reduce postoperative hypoxia, increase renal blood flow, and improve renal function during stent revascularization therapy (183). In contrast to the positive findings observed with elamipretide, outcomes of trials investigating other agents targeting mitochondrial function or related pathways have been inconsistent. For instance, a trial evaluating cyclosporine administration during the reperfusion phase of valve surgery failed to demonstrate improved outcomes, though this lack of efficacy may be attributed to suboptimal timing or dosing regimens. Additionally, smaller-scale studies have explored the potential of other interventions, such as ascorbate (Vitamin C) and thiamine: in one single-center study, administration of high-dose intravenous Vitamin C during cardiopulmonary bypass (CPB) was found to reduce the formation of neutrophil extracellular traps and was associated with enhanced lung function and shorter intensive care unit (ICU) stays (184).

Overall, the ultimate goal is reduced organ injury (myocardial infarction, stroke, AKI, *etc.*) and improved survival or faster recovery. Cardiac surgery mortality is relatively low these days (~1-3% for routine cases), so studies focus on complications. With regard to neurologic outcomes, POCD (postoperative cognitive dysfunction) is a concern after cardiac surgery (discussed further in brain section). Some evidence suggests modulating inflammation (*e.g.*, with Dex or statins) also lowers delirium incidence, as noted with Dex reducing delirium in a meta-analysis (136).

In summary for cardiac surgery: robust evidence links mtDAMPs and immune activation to postoperative complications (13, 14, 34). Interventions targeting these pathways (*e.g.*, TLR9 inhibition, antioxidants, Dexmedetomidine) have shown biochemical and some clinical improvements, though large trials sometimes yield neutral results, reminding us of the complexity of human patients. Nonetheless, strategies like using Dex or potentially remote ischemic preconditioning have been adopted in some protocols due to their low risk and potential benefit. The cardiac surgery arena continues to be one where translational research on immunometabolism is very active, as small gains (like reducing AKI by a few percentage points) can benefit thousands of patients annually.

### 4.2 Neurosurgery and brain health (neuroinflammation and cognitive outcomes)

Patients undergoing major surgery often experience neurological sequelae ranging from acute delirium to longer-lasting POCD. These phenomena are particularly common in older patients. While neurosurgeries (brain or spine operations) have direct neural impact, even non-neurosurgical procedures can affect the brain *via* systemic inflammation. Immune activation and mitochondrial dysfunction in both central and peripheral immune cells (like microglia in the brain and monocytes in blood) are implicated in these cognitive complications (64).

#### 4.2.1 Neuroinflammation after surgery

Surgical trauma induces a rise in pro-inflammatory cytokines (IL-6, TNF-α) that can cross the blood-brain barrier or signal *via* vagal pathways to activate brain endothelium and microglia (68, 72). Microglia – the resident immune cells of the brain – respond by producing their own cytokines and reactive oxygen species, leading to a state of neuroinflammation. In aged animals, surgery (like abdominal exploration under anesthesia) has been shown to activate microglia and cause synaptic loss in the hippocampus, correlating with memory deficits (64). Mitochondria are central in this: activated microglia undergo metabolic shift to glycolysis and produce ROS. Surgery and anesthesia can cause microglial mitochondrial dysfunction – one study demonstrated that sevoflurane anesthesia in young mice reduced microglial mitochondrial membrane potential and ATP, impairing their debris-clearing function (28, 128, 129). In older mice, this led to accumulation of toxic proteins and persistent inflammation.

#### 4.2.2 POCD and mitochondrial mechanisms

POCD is characterized by subtle but measurable decline in cognitive performance weeks to months after surgery. A 2024 narrative review by Zhang et al. summarized that “surgery and anesthesia can inhibit mitochondrial respiration, reduce ATP production, promote mitochondrial fission, cause Ca^2+^ dysregulation, and increase oxidative stress in the brain” – all of which can lead to impaired synaptic function and even neuronal apoptosis (64). Key observations include: in aged rodents undergoing surgery, there is evidence of mitochondrial swelling and cristae disruption in hippocampal neurons (64); increased markers of oxidative damage in brain tissue (like malondialdehyde, 4-HNE); and reduced activity of mitochondrial enzymes (complex I and IV). Also, iron accumulation in mitochondria has been observed after isoflurane anesthesia, contributing to ferroptosis (an iron-dependent form of cell death) in neurons (185). Inhibition of this pathway (using the ferroptosis inhibitor ferrostatin-1 or deferoxamine) preserved cognitive function in those models (186). Mitochondrial dynamics are altered too: excessive fission (fragmentation) of mitochondria in neurons and microglia is noted after surgery, which is usually a sign of stress and often precedes cell death (187, 188). Pharmacologically, Mdivi-1 (mitochondrial fission inhibitor) given to rodents prevented synaptic loss and cognitive impairment following sevoflurane exposure (189).

In terms of peripheral contributions, surgical patients often have increased exhausted T-cells and monocytes that secrete pro-inflammatory mediators affecting the brain. High postoperative IL-6 and delirium are strongly correlated (68, 72). Mitochondrial dysfunction in these immune cells (for instance, monocytes with low HLA-DR and low spare respiratory capacity) might predispose to an insufficiently regulated immune response that injures the brain.

#### 4.2.3 Clinical insights and interventions

Dexmedetomidine: We keep returning to Dex, but it has shown a notable reduction in delirium incidence across various surgeries, including after neurosurgery and cardiac surgery (136). The proposed mechanism is Dex’s reduction of neuroinflammation: it suppresses surgery-induced microglial activation and upregulates mitophagy in the hippocampus. In aged rats, Dex administration during anesthesia reduced IL-1β and IL-6 in the brain and improved performance in memory tasks (79). It also preserved activities of mitochondrial enzymes and prevented the drop in synaptic protein expression that was seen in controls. In clinical settings, when dexmedetomidine is used for sedation in intensive care unit (ICU) patients, it is associated with a lower incidence of delirium compared to benzodiazepines, and this finding is supported by multiple studies. For instance, a randomized controlled trial comparing dexmedetomidine and lorazepam in adult mechanically ventilated ICU patients found that dexmedetomidine allowed patients to have more delirium-free time (190). Additionally, some studies have developed delirium prevention artificial intelligence models (AID) based on reinforcement learning to optimize the dosage of dexmedetomidine; in relevant cohorts, the estimated performance yield of this model was superior to the clinical decision-making strategy employed by clinicians, enabling it to assist in clinical decisions for preventing delirium in ICU patients (191). The reason why dexmedetomidine can achieve such an effect is partly due to its ability to reduce systemic inflammatory responses, and it may also be related to its role in directly transmitting neuroprotective signals through the α2A receptors in the locus coeruleus.
Anesthetic technique: Trials comparing anesthetic types found that TIVA (total IV anesthesia) *vs.* inhalational made little difference in POCD at 3 months, but some data suggests that light depth of anesthesia (avoiding too deep anesthesia, as measured by BIS monitors) is associated with less POCD, possibly because overly deep anesthesia can cause more hypotension and hypoxia which injure mitochondria. Also, using shorter-acting drugs to avoid prolonged drug metabolism time in older patients is recommended (reduce exposure of brain to agents that may perturb mitochondria).
Anti-inflammatory strategies: Small trials of NSAIDs or steroids to reduce surgery inflammation have seen mixed results on delirium/POCD. NSAIDs have shown a relatively clear positive effect in reducing the incidence of postoperative delirium. A meta - analysis involving 8 studies with a total of 1238 participants showed that the incidence of postoperative delirium in the NSAIDs group was 11%, which was significantly lower than 19% in the control group. At the same time, they can also alleviate postoperative pain and reduce the consumption of opioid drugs (192). However, the effects of steroid drugs are controversial. The use of glucocorticoids (such as dexamethasone) in cardiac surgery does not significantly reduce the delirium rate but increases the risk of myocardial injury (193); yet, some studies have shown that a single preoperative dose of dexamethasone seems to have long - term preventive potential for POCD after cardiac surgery. For example, a 4 - year follow - up study of a randomized controlled trial found that the incidence of POCD in the dexamethasone group was lower than that in the placebo group on the 6th day after surgery and 4 years after surgery. However, due to factors such as the relatively small sample size, this study did not reach statistical significance, and the relevant conclusions still need to be further confirmed by more large - scale multicenter studies (194).
Neuroprotective agents: ARDS again appears, as it has sedative properties and regulates circadian rhythm (which if disrupted can worsen delirium). Trials giving melatonin nightly after surgery have had varied success; some show reduced delirium incidence in elderly hip fracture patients, others not (195). But melatonin’s antioxidant action in brain mitochondria is a plus. Acetyl-L-carnitine, a compound that aids mitochondrial β-oxidation and reduces oxidative stress, has been used in cognitive protection trials for cardiac surgery (196). In patients with cardiovascular disease complicated by moderate cognitive impairment, the treatment group received intravenous L-carnitine followed by oral acetyl-L-carnitine in addition to basic treatment, and its cognitive indicators were better than those of the group receiving only basic treatment, with no adverse reactions. Erythropoietin (EPO) can exert neuroprotective effects during the perioperative period, which can be achieved through methods such as reducing cell apoptosis; a study on cardiac surgery found that EPO indeed reduced some brain lesions shown by brain MRI and exerted a protective effect on the central nervous system of patients undergoing open-heart surgery (197).
Cognitive prehab and exercise: In the field of cognitive prehabilitation, exercise serves as a key “tool” for maintaining and improving mitochondrial function (198). It not only enhances mitochondrial function in skeletal muscle and other organs, increases mitochondrial content, boosts the transcriptional activity of related proteins, and reduces the production of ROS, but also improves metabolic flexibility—all of these effects lay a solid physiological foundation for the core goal of cognitive prehabilitation, which is to preserve cognitive health.

This beneficial effect of exercise on mitochondria applies to various populations, including healthy individuals, patients with chronic diseases, and the elderly. However, when incorporating exercise into cognitive prehabilitation programs to optimize mitochondrial and metabolic function, it is necessary to tailor the exercise dosage and duration according to individual conditions.

Minimizing bypass and emboli: In cardiac surgery, using off-pump techniques or improved filters can reduce micro-emboli to brain which cause inflammation and mitochondrial damage. Fewer emboli correlates with less cognitive decline.
Targeting ferroptosis: The recognition that iron-mediated oxidative damage (ferroptosis) plays a role in POCD has led to exploring iron chelators. In rodent models, deferoxamine given before or right after surgery preserved cognition (136). There is a trial in orthopedic surgery testing whether low-dose deferoxamine infusion can reduce delirium incidence in elderly (since they often have high brain iron content).

Postoperative delirium typically occurs in first few days; POCD is evaluated at 1–3 months. Delirium has clear associations with increased mortality at 1 year and risk of long-term cognitive decline. The multi-factorial nature of delirium means addressing one pathway (like just IL-6 or just mitochondria) may not wholly prevent it, but any reduction is valuable. Some centers have integrated multi-component interventions (*e.g.*, Dex sedation, early mobilization, cognitive stimulation, good pain control with regional anesthesia to avoid high opioid doses – all of which incidentally benefit mitochondria by reducing stress) and reported lower delirium rates.

In summary for neurological outcomes: immune cell and neural cell mitochondrial dysfunction is strongly linked to postoperative cognitive disorders. Interventions that preserve mitochondrial function (like Dex, melatonin, avoiding deep anesthesia) show promise in mitigating delirium and POCD. More research is ongoing to pinpoint the optimal neuroprotective regimen. A big challenge is patient heterogeneity – age, baseline cognitive reserve, *etc.*, so future approaches may combine therapies (antioxidant + anti-inflammatory + environmental mods) personalized to risk profiles.

### 4.3 Liver and kidney surgeries (ischemia–reperfusion injury and immune modulation)

#### 4.3.1 Liver surgery/transplantation

The liver is rich in macrophages (Kupffer cells) and is often subject to IR during inflow occlusion in resection or during transplant graft implantation. Hepatic IR injury is a significant issue – it can lead to primary graft dysfunction in transplants or liver failure after major resection. Mechanistically, upon reperfusion, Kupffer cells are activated and produce large amounts of ROS and cytokines (TNF, IL-1) that kill hepatocytes. Mitochondrial dysfunction in Kupffer cells plays a central role: IR causes Kupffer cell mitochondria to release mtDNA and ROS, activating NLRP3 inflammasomes which then release IL-1β that amplifies neutrophil recruitment (67). There is direct evidence: in a mouse model, PINK1 overexpression (enhancing mitophagy) in Kupffer cells reduced their mtDNA release and NLRP3 activation, resulting in markedly less liver IR injury (67). Similarly, mice lacking NLRP3 or treated with an IL-1 receptor antagonist had smaller rises in ALT/AST and better histology after liver IR (199). Another pathway is cGAS-STING in liver non-parenchymal cells responding to mtDNA; a study titled “Blockade of mtDNA release ameliorates hepatic IR through avoiding cGAS-STING activation” demonstrated that using a pharmacological inhibitor of Bax/Bak (which blocks mtDNA release from mitochondria) reduced liver injury and that adding a STING antagonist likewise protected the liver (51).

Clinically, strategies to mitigate IR include ischemic preconditioning (clamping the portal triad briefly before a long ischemia), which has been shown to lower postoperative transaminases and reduce cytokine release. Pharmacologically, N-acetylcysteine (NAC) is often given during liver transplant reperfusion to provide glutathione substrate – some trials show improved early graft function with NAC, attributed to decreased oxidative mitochondrial damage. Sodium nitrite administered before reperfusion has shown promise in reducing liver IR injury by S-nitrosating mitochondrial proteins and attenuating ROS. For example, in a phase II trial, liver transplant patients given low-dose nitrite had lower AST release and better bile production.

Mitochondrial transplantation has even been tested in liver: a study on male Yorkshire pigs with left hepatic ischemia-reperfusion injury showed that portal vein infusion of autologous mitochondria significantly reduced liver damage, improved liver function (such as lowering AST and inflammatory factors, promoting lactate clearance), and exhibited potential for clinical liver transplantation (200).

Another aspect is steatotic livers (fatty livers) which are more susceptible to IR due to baseline mitochondrial dysfunction. Such livers in surgery have higher rates of failure. Approaches like perfusion machine pre-treatments adding agents like *trimetazidine* (a metabolic modulator) aim to improve mitochondrial fatty acid oxidation during preservation, thus reducing IR damage when implanted.

#### 4.3.2 Kidney surgery/transplantation

The kidney, especially proximal tubules, are rich in mitochondria and highly vulnerable to ischemia. Cardiac surgery, as noted, often causes AKI *via* inflammatory and mitochondrial pathways. In kidney transplantation, ischemia time and donor factors dictate initial graft function. Studies find that urinary mtDNA is elevated in both native kidney AKI and transplant delayed graft function (201). In severe trauma, high plasma mtDNA predicted later development of AKI (202).

Interventions in renal IR revolve around either reducing metabolic demand or enhancing mitochondrial repair: hypothermia is protective in kidney (surgeons often externally cool kidneys during temporary occlusion if possible, as lower temperature slows mitochondrial respiration and ROS). Ischemic preconditioning and postconditioning (short cycles of ischemia right at reperfusion start) have shown benefits in animal kidneys, linked to preservation of mitochondrial ultrastructure. Some agents: erythropoietin and MitoQ have shown protective effects in experimental models of renal IR (203, 204).

One breakthrough recently is in machine perfusion of kidneys: adding melatonin to the preservation solution improved outcomes in pig transplants, likely due to melatonin’s mitochondrial protection in tubular cells (less ROS, better ATP). Also, attempts to use *ex vivo* gene therapy during machine perfusion (like delivering an Nrf2 gene or small interfering RNAs against pro-apoptotic factors into the kidney) are underway, which if successful could drastically reduce IR injury.

#### 4.3.3 Clinical outcomes and trials

Liver transplantation is seeing improvement in early allograft function partly thanks to these targeted interventions; for instance, normothermic machine perfusion (which keeps mitochondria energized) significantly reduces AST release and graft injury compared to static cold storage. A trial of cyclosporine at reperfusion in liver transplant showed a trend to less AST release, though not significant. In AKI after cardiac surgery, despite many candidate therapies, the incidence remains high ~20%. However, some hospitals report reduced AKI with protocols including high-dose statin pre-op, avoiding deep hypothermia, using Dex, and tight glycemic control – all measures that have mitochondrial rationales.

In summary, for liver/kidney surgeries, immune cell mitochondria (especially Kupffer cells and infiltrating monocytes) orchestrate a lot of the IR injury *via* inflammasomes and cytokine release. Strategies that preserve mitochondrial integrity (ischemic preconditioning, antioxidants, mitophagy enhancers) or temper immune activation (IL-1 blockade, TLR9 inhibition) show beneficial effects in reducing organ injury. Overcoming translational hurdles (optimal timing, dosage) is still ongoing, but some interventions (*e.g.*, NAC for liver, remote preconditioning, *etc.*) have already made it into practice due to favorable risk-benefit profiles.

### 4.4 Integrated clinical insights and cross-organ implications

To consolidate the clinical evidence: across heart, brain, liver, and kidney domains, there is a unifying theme that mitochondrial dysfunction in immune and parenchymal cells contributes to perioperative organ injury, and conversely, that interventions targeting mitochondrial preservation or immune modulation correlate with improved outcomes (even if modestly in some cases) (28, 64).

The clinical data underscores both the potential and the challenge: while mechanistic studies are convincing and smaller trials encouraging, large randomized trials sometimes fail to meet endpoints, reminding us of patient complexity and the need to target the right patients. This is where precision medicine approaches (as touched on) will likely come into play – identifying which patients have immune mitochondrial dysfunction *via* biomarkers and tailoring therapies accordingly.

## 5 Challenges and future directions

Despite compelling mechanistic insights and promising early-phase interventions, there are several challenges in translating perioperative mitochondrial immunomodulation into standard clinical care:

### 5.1 Timing and deliverability

Many mitochondrial-protective strategies are highly time-sensitive. The perioperative period, especially in emergencies, often doesn’t allow much pre-planning. Interventions like ischemic preconditioning or certain drugs must be given before or during the injurious event to be effective. Identifying the optimal timing (preoperative dose *vs.* intraoperative infusion *vs.* immediate postoperative) for each therapy is critical. For example, IL-1 blockade might need to be present right at reperfusion to prevent the initial inflammasome burst. If given too late (after cytokines already surged), the benefit wanes. Likewise, mitochondrial transplantation likely offers greatest benefit if done within minutes of reperfusion – logistically challenging but possibly achievable in controlled surgical settings (like transplant surgery where one can plan to inject mitochondria into the graft portal vein just before unclamping). Solutions include developing drugs that can be easily and rapidly administered (like an inhaled mitochondrial antioxidant that could be given while the patient is being induced) or modular “kits” for mitochondrial isolation in the OR for autologous transplant.

### 5.2 Patient heterogeneity

Surgical patients vary widely in age, comorbidities (diabetes, heart failure), and baseline immune status. These factors influence mitochondrial health [*e.g.*, older patients have more mitochondrial DNA mutations and less antioxidant capacity (113)]. The same intervention might have different efficacy across patient profiles. A therapy that works in a young healthy patient to blunt inflammation might not in an older patient with immunosenescence (where the immune cells might not respond vigorously anyway – in fact, boosting their mitochondria might be more needed to avoid immunosuppression). Therefore, personalized medicine is crucial. Biomarkers such as mitochondrial function assays in blood immune cells (like measuring mitochondrial oxygen consumption in isolated monocytes), plasma mtDNA levels, or inflammatory gene expression panels can stratify patients into phenotypes (hyper-inflammatory *vs.* hypo-inflammatory). Future trials will likely incorporate such stratification. For instance, a trial of an IL-6 inhibitor could target patients identified as having a high IL-6 genotype or high pre-op IL-6 levels, to enrich for those likely to benefit.

### 5.3 Safety and off-target effects

Modulating the immune system and mitochondria can have unintended consequences. Complete suppression of inflammation can impair wound healing or increase infection risk, *e.g.*, while IL-1 or TNF blockers might reduce sterile inflammation, they could predispose to infection in the immune-compromised postoperative host. Similarly, drugs like cyclosporine that target mPTP are not entirely specific to injured cells – they might prevent desired apoptosis of severely damaged cells, potentially leading to necrosis instead.

In addition, therapies like mitochondrial transplantation (if not autologous) must avoid immune reactions and prevent imported mitochondria from fueling cancer cells (they may aid cancer survival in hypoxia with micrometastases). Their safety needs extensive research. For instance, mitochondrial augmentation therapy (MAT) for children with single large - scale mitochondrial DNA deletion syndromes (SLSMDs) showed no serious side effects, no immune rejection, normal blood/metabolism, and higher mtDNA—but this is preliminary (205). Wider use requires more long-term studies across diseases to verify safety, effectiveness and risks.

Gene therapies also carry risks, such as the potential for long-term off-target effects or germline transmission. However, when these therapies are administered to somatic cells in adults and only used for a short period during the perioperative period, the associated risks related to germline transmission are significantly reduced.

### 5.4 Measurability and monitoring

One cannot manage what one cannot measure. Currently, we don’t have routine bedside monitors for “mitochondrial function.” However, that might change – the mentioned COMET device is a fiber-optic probe that measures mitochondrial oxygen tension and respiratory chain redox state non-invasively *via* an optical technique (206). It’s being tested in cardiothoracic surgery patients placed on the liver or skeletal muscle to detect mitochondrial “shock” early (207). Such devices, once validated, could guide therapy: for instance, if the probe shows high tissue oxygen but poor utilization (indicative of mitochondrial dysfunction), the team might intensify mitochondrial support interventions (208). For another example, by continuously monitoring the respiratory function of a patient’s immune cells *in vitro* (potentially using a cell metabolism assay kit, with measurements taken every few hours), if the immune cells are found to be in an “energy crisis” (*e.g.*, in cases of sepsis, the adenosine triphosphate (ATP) levels of peripheral blood mononuclear cells (PBMCs) drop sharply), an early warning can be issued promptly, followed by the initiation of immunostimulatory therapy or metabolic therapy.

### 5.5 Multi-targeted approaches

Given the complex network of inflammation, tackling one pathway might not suffice. Future strategies likely will use combination therapy – akin to bundle protocols. For example, a “mitoprotection bundle” for high-risk surgery might include: a) a sulforaphane-based supplement a day before surgery (to activate Nrf2 antioxidant responses), b) remote ischemic preconditioning during anesthesia induction (209), c) low-dose IL-1ra (anakinra) at start of reperfusion, d) postoperative Dexmedetomidine infusion, and e) high-protein nutrition with omega-3 fatty acids to support recovery. Each component addresses different facets (antioxidant, DAMPs release, cytokine signaling, sympathetic modulation, cell membrane stabilization). Testing such bundles systematically would be a challenge as it’s hard to parse which element gives benefit, but clinically, if it works, it works. Another angle is postoperative exercise or mobilization – early mobilization might stimulate mitochondrial turnover and function, aiding recovery (though too-early mobilization if patient is unstable could be harmful, so balance needed).

### 5.6 Organ cross-talk

Interventions aimed at one organ’s protection might inadvertently stress another organ’s mitochondria. For example, some drugs to protect heart mitochondria might reduce blood flow to kidney (like high-dose vasoconstrictors). So a holistic view is needed. Also, immunologic cross-talk means modulating immune cells for one organ affects systemic immunity. If one reduces Kupffer cell activation to protect the liver, could that compromise systemic pathogen clearance? Perhaps not significantly for short-term, but careful study is needed.

### 5.7 Regulatory and practical issues

Therapies like biologics or gene therapy in the perioperative space face regulatory hurdles – for instance, using an unapproved biologic (like mitochondrial transfer) would require intensive oversight and likely be limited to academic centers initially. Scaling production of mitochondria or viral vectors in a sterile way in the OR is non-trivial. Costs could be an issue – *e.g.*, CAR-T cells are extremely expensive; obviously we won’t do CAR-T for every surgical patient, but maybe something like a temporary engineered cell therapy for the ICU could be conceived if costs drop. Also training clinicians to adopt these new treatments (which might not be in the typical anesthesiologist or surgeon toolbox) requires education and demonstrations of clear benefit.

Future directions to overcome these challenges include biomarker-driven clinical trials that enrich enrollment with patients exhibiting high mtDAMPs or immune dysfunction, such as testing NLRP3 inhibitors in cardiac surgery patients with elevated preoperative neutrophil–platelet aggregates or high mtDNA levels after CPB, thereby increasing the power to detect benefits in those most likely to respond; development of safer modulators, such as next-generation inflammasome inhibitors targeted to activated immune cells *via* antibodies to activation markers, or ROS-activated prodrugs and nanoparticles to ensure localized action; leveraging genomics and AI by integrating transcriptomics with clinical and laboratory data to generate personalized recommendations (*e.g.*, GM-CSF for immune paralysis with monocyte mitochondrial support, or IL-1 blockade for hyperinflammation); implementing prophylactic metabolic conditioning in elective surgeries through dietary interventions like ketone ester drinks to elevate BHB, exercise, and pharmacological pre-conditioning with agents such as metformin or NAD+ boosters to enhance mitochondrial resilience; advancing *ex vivo* organ and cell conditioning by treating donor organs before transplantation to induce protective mitochondrial enzymes or reduce DAMPs, and exploring selective apheresis to reset immune function in critically ill patients; and focusing on long-term outcomes by assessing whether early mitochondrial and immune-targeted interventions can reduce downstream chronic complications, such as Chronic Kidney Disease(CKD) or cognitive decline, potentially through mechanisms like IL-1 blockade reducing pro-fibrotic signaling in the kidneys.

In essence, the future of perioperative organ protection is likely to be multidisciplinary and personalized, harnessing immunology, metabolism, and bioengineering. It will involve tight monitoring and rapid responses to derailing physiology, much like critical care but with more proactive augmentation of the body’s defenses (rather than only rescue). The concept of an “immune-metabolic anesthesia” or “surgical metabolic care” team may emerge, where anesthesiologists, surgeons, and immunologists work together to interpret data and adjust therapies in real-time.

As these challenges are met with innovative solutions, we edge closer to making perioperative modulation of immune cell mitochondria a standard part of surgical care, potentially transforming outcomes for high-risk surgeries and improving recovery and quality of life for patients.

## 6 Conclusion

The perioperative period represents a perfect storm of physiological stress where the immune system’s response can tip the balance between recovery and organ injury. At the heart of this response are the tiny powerhouses within immune cells – mitochondria – which orchestrate immune activation, energy production, and cell fate. Surgical ischemia, trauma, and circulatory perturbations can derail mitochondrial function in immune cells, leading to excessive DAMPs release, inflammasome activation, metabolic failure, and ultimately tissue damage and immunosuppression. This review has highlighted how mitochondrial-immune crosstalk is a unifying paradigm explaining much of perioperative organ dysfunction, from sterile inflammation in the heart and brain to delayed healing and infection susceptibility.

Encouragingly, what was once viewed as an inevitable “inflammatory cascade” of surgery now appears modifiable. A new arsenal of therapies – mitochondria-targeted antioxidants, metabolic modulators, DAMPs/inflammasome inhibitors, mitochondrial transplantation, and immunometabolic reprogramming techniques – shows promise in experimental models and early clinical trials for blunting the pathological immune response while preserving essential host defenses. The evidence reviewed from cardiac surgery, neurosurgery, and abdominal organ surgeries underscores that interventions as simple as choosing an anesthetic that preserves mitochondrial quality (*e.g.*, Dexmedetomidine) or as high-tech as infusing healthy mitochondria can translate into tangible patient benefits. Patients experienced fewer arrhythmias, less acute kidney injury, sharper cognitive recovery, and better graft function when these principles were applied, pointing to the clinical relevance of targeting immune cell mitochondria.

Despite the progress, challenges remain. The complexity of the immune response means that a fine balance must be struck – too much suppression of inflammation can be as dangerous as too little. Future approaches will need to be nuanced and patient-specific, guided by biomarkers of immune and mitochondrial status. As research continues, we anticipate development of point-of-care assays for mitochondrial function and immune activation that will enable clinicians to administer the right mitochondrial therapy to the right patient at the right time. Large-scale trials are awaited to confirm the efficacy and safety of these novel strategies (such as IL-1 antagonists in surgical SIRS or mitophagy enhancers in transplantation). Furthermore, as technologies like normothermic perfusion and cell therapy mature, they open doors to practically implement mitochondrial modulation – for instance, by treating an organ with agents that boost mitochondrial resilience before it’s ever implanted into a patient.

In conclusion, perioperative modulation of mitochondrial function in immune cells represents a paradigm shift in organ protection. It moves us from a paradigm of passively supporting failing organs to one of proactively reprogramming the body’s stress response at a cellular power-source level. By buffering the mitochondria through the surgical insult – preventing the flood of mtDAMPs, sustaining ATP production in immune cells, and averting immune cell exhaustion – we can reduce collateral tissue damage and foster a more balanced recovery response. The concept of the “inflamed mitochondrion” as both culprit and target has illuminated a new frontier of therapeutic opportunities. With multidisciplinary collaboration and innovative clinical trials, this frontier is rapidly advancing from bench to bedside.

The vision for the near future is that an array of mitochondrial therapies will be integrated into enhanced recovery protocols for surgical patients: an extra infusion in the OR here, a targeted drug there, all aimed at keeping the patient’s cellular batteries running optimally. If successful, patients will emerge from surgery with less organ dysfunction, shorter ICU stays, and improved long-term outcomes. The scientific journey to get here exemplifies the power of translational research – unraveling fundamental mechanisms of immunometabolism and then harnessing that knowledge to improve patient care. Protecting the cell’s powerhouse to shield the whole organ may soon become a guiding tenet in perioperative medicine, fulfilling the promise of precision organ protection and safer surgery for millions of patients.

## Glossary

ATP: Adenosine triphosphate
AMPK: activated Protein Kinase
ARDS: Acute respiratory distress syndrome
AKI: Acute kidney injury
CARS: Compensatory Anti-inflammatory Response Syndrome
cGAS: Cyclic GMP-AMP synthase
cGAMP: Cyclic GMP-AMP
CPB: Cardiopulmonary bypass
CRS: Cytokine Release Syndrome
CKD: Chronic Kidney Disease
DAMPs: Damage-associated molecular patterns
MARS: Mixed Antagonist Response Syndrome
mtDAMPs: Mitochondrial damage-associated molecular patterns
mtDNA: Mitochondrial DNA
MyD88: Myeloid differentiation primary response 88
MAS: Macrophage Activation Syndrome
mPTP: mitochondrial permeability transition pore
MDSCs: Myeloid-derived suppressor cells
mTORC: mammalian Target of Rapamycin Complex
NF-κB: Nuclear factor kappa-light-chain-enhancer of activated B cells
NAD+: Nicotinamide Adenine Dinucleotide
NLRP3: NLR family pyrin domain containing 3
NR4A: Nuclear receptor subfamily 4 group A
ROS: Reactive oxygen species
RNS: Reactive Nitrogen Species
STING: Stimulator of interferon genes
TLR9: Toll-like receptor 9
TOX: Thymocyte selection-associated high mobility group box protein
IRF3: Interferon regulatory factor 3
TBK1: TANK-binding kinase 1
IL-1β: Interleukin-1 beta
IL-6: Interleukin-6
IL-1: Interleukin-1
TNF-α: Tumor necrosis factor-alpha
IFN: Interferon
SIRS: Systemic inflammatory response syndrome
IR: Ischemiareperfusion
ICU: Intensive care unit
the cGAS-STING pathway: Cyclic GMP-AMP synthase–stimulator of interferon genes pathway
PD-1: Programmed cell death protein 1
PD-L1: Programmed cell death ligand 1
POCD: Postoperative cognitive dysfunction
